# Burden of Breast Cancer Attributable to a Diet High in Red Meat at Global, Regional, and National Levels: An Analysis of the Global Burden of Disease Study 2021

**DOI:** 10.34172/aim.34079

**Published:** 2025-05-01

**Authors:** Fang Zhou, Jincheng Tang, Renyi Yang, Puhua Zeng, Jianxiong Cao

**Affiliations:** ^1^Hunan Provincial Hospital of Integrated Traditional Chinese and Western Medicine, Hunan University of Chinese Medicine, Changsha, Hunan, China; ^2^Institute of Traditional Chinese Medicine Oncology, Hunan Academy of Chinese Medicine, Changsha, Hunan, China; ^3^The First Hospital of Hunan University of Chinese Medicine, Changsha, Hunan, China

**Keywords:** Breast cancer, Diet high in red meat, Disability-adjusted life years, Global Burden of Disease Study, Sociodemographic index

## Abstract

**Background:**

Dietary factors are a key risk for breast cancer. This study examines the global burden of breast cancer attributed to a high red meat diet from 1990 to 2021.

**Methods:**

Using Global Burden of Disease Study (GBD) 2021 data, deaths and disability-adjusted life-years (DALYs) were analyzed globally, regionally, and nationally. Trends were assessed through estimated annual percentage changes (EAPCs) in age-standardized mortality (ASMR) and DALY (ASDR) rates. A decomposition analysis quantified the contributions of population growth, aging, and epidemiological changes. The relationship between sociodemographic index (SDI) and burden was examined using Spearman rank test. Health inequalities were assessed using the Slope Index of Inequality for absolute inequality and the Concentration Index for relative inequality.

**Results:**

By 2021, breast cancer deaths and DALYs linked to high red meat intake had increased significantly compared to 1990, despite a decline in ASMR [EAPC: -0.77 (95% CI -0.82 to -0.72)] and ASDR [EAPC: -0.65 (95% CI -0.70 to -0.60)]. These trends were driven by population growth and aging, with regional variability in the pace of demographic transitions. North Africa and the Middle East experienced the largest rise in ASMR [EAPC: 2.03 (95% CI 1.79 to 2.26)], while Pacific Island nations had the highest ASMR and ASDR. High-SDI regions had the highest ASMR [1.14 per 100000 (95% UI -0.01‒2.43)] and ASDR [33.07 per 100000 (95% UI -0.02‒69.90)], with a positive SDI-burden correlation in low- and middle-SDI regions (*P*<0.05), but a negative correlation in high-SDI regions (*P*<0.05). From 1990 to 2021, absolute inequality [35.79 (95% CI 29.13‒42.46) vs. 4.99 (95% CI -1.59-11.56)] and relative inequality [0.18 (95% CI 0.16‒0.21) vs. 0.02 (95% CI -0.01‒0.05)] decreased.

**Conclusion:**

Although ASMR and ASDR have declined, the absolute burden of breast cancer due to high red meat intake remains significant, particularly in aging and rapidly urbanizing populations. Policy interventions should include taxation on red meat, restrictions on processed meat, and public health campaigns promoting dietary modifications. Targeted screening programs in high-risk regions, especially for middle-aged and elderly populations, are critical for mitigating the future disease burden.

## Introduction

 Breast cancer represents a major risk and challenge to women’s health worldwide. In 2022, it accounted for a notable proportion of newly diagnosed malignancies and cancer-related fatalities, ranking as the fourth leading cause of cancer mortality and the second most common cancer.^1^ Approximately 0.5% to 1% of breast cancer cases occur in men, with a lifetime risk of about 1 in 1000, and the incidence increases with age.^2^ Beyond its medical implications, breast cancer imposes a significant financial burden, especially in low-income countries, where the financial toxicity rate is estimated to be as high as 78.8%.^3^

 Breast cancer is a multifactorial disease influenced by genetic predisposition, hormonal fluctuations, lifestyle choices, and environmental factors. Among lifestyle factors, dietary habits have drawn increasing attention due to their modifiability. Unhealthy diets, particularly those high in processed foods, alcohol, and red meat, have been investigated for their potential role in increasing breast cancer risk.^4^ Studies suggest that the consumption of red meat may be linked to higher breast cancer incidence, possibly attributed to its saturated fats, heme iron, and carcinogenic compounds formed during high-temperature cooking.^5^ Additionally, alcohol intake and diets high in refined sugars have been identified as potential risk factors for breast cancer development.^6,7^

 Epidemiological studies have attempted to quantify the link between breast cancer risk and red meat intake. An analysis using a random-effects model revealed a significant positive correlation between higher red meat intake and increased risk of breast cancer [risk ratio per 100 g/d, 1.10].^8^ Moreover, a prospective cohort study reported that individuals with high red meat consumption had a 23% increased risk of developing invasive breast cancer.^9^ Dietary patterns may influence breast cancer prognosis. Studies suggest that survivors adhering to diets rich in vegetables and fruits experience a 15% to 43% reduced risk of breast cancer-specific mortality compared to those following a Western diet.^10^ However, it is essential to acknowledge that other confounding variables, including overall diet quality, physical activity, body mass index, and hormonal factors, could influence this relationship.

 Although prior research has examined breast cancer epidemiology at global and regional levels,^11,12^ the burden specifically linked to high red meat consumption remains inadequately characterized. The Global Burden of Disease (GBD) 2021 dataset provides a robust framework for assessing behavioral and metabolic risk factors linked to breast cancer.^13^ In 2021, dietary factors, including high red meat intake, were estimated to account for 12.07% of breast cancer-related deaths, exceeding several other risk determinants (Figure S1). To provide deeper insight into long-term epidemiological trends and inform policy strategies, this study evaluates the global, national, and regional burden of breast cancer associated with high red meat consumption from 1990 to 2021 using GBD 2021 data.

## Materials and Methods

###  Data Collection

 The GBD study is one of the most extensive epidemiological projects worldwide, designed to quantitatively evaluate health burdens across various timeframes and geographic regions.^14^ This analysis utilized data from the Global Health Data Exchange query tool (https://ghdx.healthdata.org/gbd-2021), where “disability-adjusted life years (DALYs)” and “deaths” served as outcome measures, while “number” and “rate” were used as key metrics. The primary risk factor examined was “diet high in red meat,” with “breast cancer” designated as the associated cause. The study relied on publicly available, de-identified data, eliminating the need for institutional review board approval.^15^

###  Definitions

 GBD 2021 defines breast cancer using the International Classification of Disease (ICD)-9 codes (174‒175.9, 217‒217.8, 233.0, 238.3, 239.3, 610‒610.9) and ICD-10 codes (C50-C50.9, D05-D05.9, D24-D24.9, D48.6, D49.3). In terms of risk factor classification, GBD 2021 sets a theoretical minimum risk exposure level (TMREL). A high-red-meat diet falls under the dietary factors subcategory of behavioral risks, with its TMREL defined as 0–200 g/d of unprocessed red meat (muscle meat from mammals such as lamb, pork, beef, etc).^13^

 The GBD database uses deaths and DALYs as metrics to assess the disease burden. Deaths reflect the direct health impact of diseases, while DALYs include both years lived with disability and years of life lost, capturing both non-fatal and fatal health consequences to offer a thorough measure of overall disease burden.^14^

 The sociodemographic index (SDI) used in the GBD study quantifies the socioeconomic development of countries. It is derived from lag-distributed income per capita, average years of schooling for individuals aged 15 and older, and the total fertility rate for those under 15.^16^ Additionally, age is segmented into 20 groups, each spanning a 5-year interval, enabling age-stratified analysis of disease epidemiology.

###  Statistical Analyses

 The GBD database uses Bayesian hierarchical models and the Cause of Death Ensemble model to infer and adjust statistical data, accounting for regional variability and missing data. These models incorporate multiple sources of data and apply prior distributions to handle uncertainty in the data, including missing values due to regional sparsity or inconsistent reporting. While these models are robust, they rely on a limited assumption about data consistency across regions, and potential biases from underreporting in low-income countries may affect the estimations. These potential biases should be considered when interpreting the results, especially in regions where health reporting infrastructure is less developed.^17,18^

 The age-standardized rate (ASR) is a key indicator for eliminating the impact of differences in population age structure on disease burden. These indicators facilitate cross-regional comparisons of health losses. Age-standardized mortality rates (ASMRs), age-standardized DALY rates (ASDRs), deaths, and DALYs are reported with a 95% uncertainty interval (UI), derived from the 25^th^ and 975^th^ percentiles of 1000 simulations within the uncertainty distribution. Additionally, the estimated annual percentage change (EAPC) is utilized to assess trends in ASR over time. The calculation formula for EAPC is EAPC = (exp(*β*) – 1) × 100, where *β* is the regression coefficient derived from a linear regression of ASR over time. A positive lower bound of the 95% confidence interval (CI) for EAPC indicates an increasing trend, while a negative upper bound suggests a downward trend. If the 95% CI spans zero, no statistically significant change is inferred. Specific formulas for ASR and EAPC have been reported in previous literature.^16,19^ The distinction between UIs and CIs is that UIs account for both data uncertainty and model variability, while CIs reflect the precision of the EAPC estimates within the regression framework

 Through decomposition analysis of the data, we quantified the effects of epidemiological changes, population growth, and aging on the burden of breast cancer associated with high red meat consumption, evaluating the contribution of each factor to disease burden changes.^20^ Lag effects of dietary changes on breast cancer incidence and mortality were also considered. While the temporal trends captured in the GBD study are valuable, the full long-term effects of dietary habits may not be completely reflected due to the inherent lag in the development of chronic diseases like breast cancer. This limitation should be noted, as changes in diet may take decades to manifest in disease burden estimations.

 Furthermore, the Spearman rank test was used to identify nonlinear correlations between disease burden indicators (ASR, EAPC) and SDI.^21^ Cross-country inequality analysis was also conducted to evaluate disparities in disease burden related to SDI. The slope index of inequality was derived by regressing the midpoint of the cumulative population distribution across SDI levels against the DALY rate, providing a measure of absolute inequality. The concentration index was used to assess relative inequality by applying the Lorenz curve to the cumulative proportion of DALYs and population distribution across SDI levels, with numerical integration used to calculate the area under the curve.^22^ Furthermore, frontier analysis was used to evaluate the relative efficiency and potential for improvement in disease control across different SDI levels, quantifying disease burden performance and relative disparities at various SDI levels.^23^ Data analysis and visualization were conducted using the R software (version 4.3.2) and Rstudio, with a significance threshold of *P* < 0.05.

## Results

###  Deaths and DALYs at the Global Level

 Globally, in 2021, breast cancer deaths and DALYs attributable to a diet high in red meat were estimated at 81 506.23 (95% UI -25.57–175 444.92) and 2 451 718.64 (95% UI -790.88–5 232 217.29), respectively, showing an increase of 80.83% (95% UI 30.77%–184.34%) and 75.52% (95% UI 31.26%–162.97%) compared to 1990. Notably, negative values in the UIs for both deaths and DALYs are the result of model adjustments where some data points suggest a potentially beneficial impact of high red meat intake on breast cancer, which leads to biologically implausible negative estimates. These negative values should be interpreted as statistical uncertainty, not as an actual protective effect.

 In 2021, the ASMR and ASDR were 0.96 per 100 000 (95% UI -0.01–2.06) and 28.37 per 100 000 (95% UI -0.01–60.54), respectively, with EAPCs from 1990 to 2021 of -0.77 (95% CI -0.82 to -0.72) and -0.65 (95% CI -0.70 to -0.60), both demonstrating a consistent decrease. Notably, while the burden of breast cancer deaths and DALYs attributable to a diet high in red meat was significantly higher in women than men, the relative increase in male breast cancer deaths and DALYs exceeded that observed in women. During this period, the ASMR [EAPC: 0.63 (95% CI 0.52 to 0.73) vs. -0.73 (95% CI -0.78 to -0.68)] and ASDR [EAPC: 0.84 (95% CI 0.73 to 0.96) vs. -0.65 (95% CI -0.71 to -0.60)] for men exhibited an upward trajectory, whereas for women, they declined (Table 1).

**Table 1:** Global Deaths and DALYs of Breast Cancer Attributed to High Red Meat Diet from 1990 to 2021

**Year**	**Both**	**Female**	**Male**
1990			
Deaths (95% UI)	45073.85 (-13.31‒96485.06)	44491.95 (-13.16‒95186.57)	581.90 (-0.17‒1282.01)
DALYs (95% UI)	1396840.46 (-435.82‒3004079.75)	1379721.38 (-431.20‒2965445.61)	17119.08 (-4.80‒37498.68)
ASMR/100 000 persons (95% UI)	1.17 (-0.01‒2.50)	2.11 (-0.01‒4.52)	0.03 (-0.01‒0.07)
ASDR/100 000 persons (95% UI)	33.31 (-0.01‒71.68)	63.22 (-0.02‒135.90)	0.88 (-0.01‒1.92)
2021			
Deaths (95% UI)	81506.23 (-25.57‒175444.92)	79956.96 (-25.28‒172077.10)	1549.27 (-0.36‒3495.83)
DALYs (95% UI)	2451718.64 (-790.88‒5232217.29)	2407092.26 (-782.02‒5134048.49)	44626.38 (-10.45‒101061.73)
ASMR/100 000 persons (95% UI)	0.96 (-0.01‒2.06)	1.76 (-0.01‒3.78)	0.04 (-0.01‒0.09)
ASDR/100 000 persons (95% UI)	28.37 (-0.01‒60.54)	53.95 (-0.02‒115.10)	1.07 (-0.01‒2.43)
1990-2021			
PC of deaths (%)	80.83 (30.77‒184.34)	79.71 (29.64‒193.36)	166.24 (26.72-341.30)
PC of DALYs (%)	75.52 (31.26‒162.97)	74.46 (30.11‒165.32)	160.68 (6.34-332.51)
EAPC of ASMR (95% CI)	-0.77 (-0.82 to -0.72)	-0.73 (-0.78 to -0.68)	0.63 (0.52 to 0.73)
EAPC of ASDR (95% CI)	-0.65 (-0.70 to -0.60)	-0.65 (-0.71 to -0.60)	0.84 (0.73 to 0.96)

DALYs, Disability-adjusted life-years; ASMR, Age-standardized mortality rate; ASDR, Age-standardized disability-adjusted life-year rate; PC, Percentage change; EAPC, Estimated annual percentage change; UI, Uncertainty interval; CI, Confidence interval.

###  Deaths and DALYs at the Regional Level

**Figure 1:**
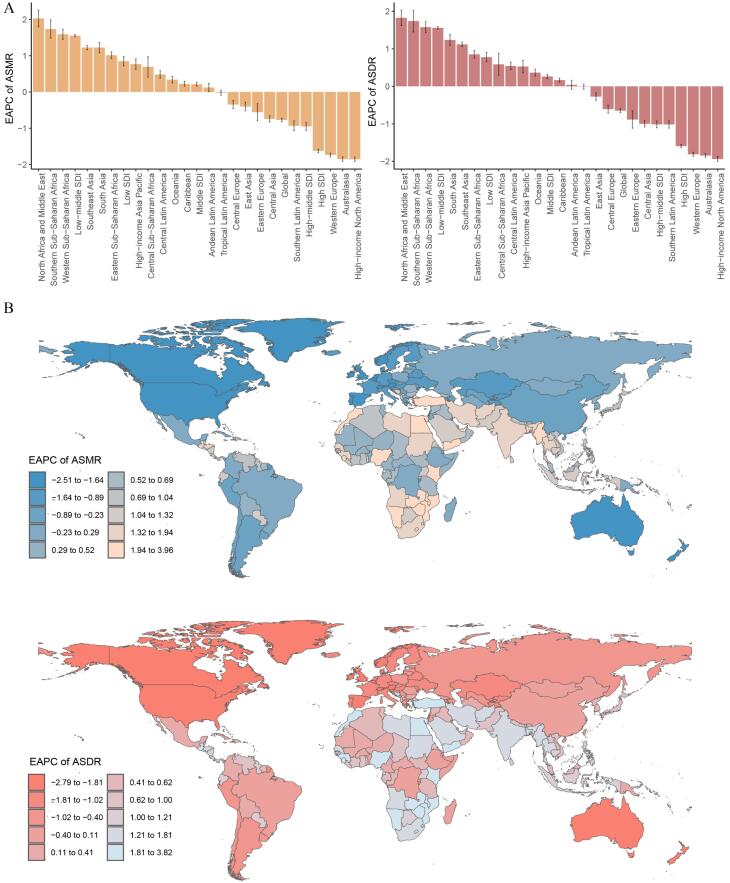
EAPCs of ASRs for Breast Cancer Attributed to Diet High in Red Meat from 1990 to 2021. **A.** EAPCs of ASMR and ASDR across 21 regions and 5 SDI regions. **B.** EAPCs of ASMR and ASDR across 204 countries and territories. EAPCs, Estimated annual percentage changes; ASRs, Age-standardized rates; ASMR, Age-standardized mortality rate; ASDR, Age-standardized disability-adjusted life-year rate; SDI, Sociodemographic index

 In 2021, Southern Sub-Saharan Africa had the greatest ASMR [2.01 per 100 000 (95% UI -0.01–4.38)] and ASDR [55.65 per 100 000 (95% UI -0.02–121.23)] for breast cancer attributable to high red meat intake. From 1990 to 2021, 13 regions exhibited a positive EAPC in both ASMR and ASDR, while 8 regions had a negative EAPC. North Africa and the Middle East experienced the greatest upward trend in ASMR [EAPC: 2.03 (95% CI 1.79 to 2.26)] and ASDR [EAPC: 1.83 (95% CI 1.62 to 2.03)], whereas High-income North America demonstrated the most pronounced downward trend in ASMR [EAPC: -1.86 (95% CI -1.92 to -1.79)] and ASDR [EAPC: -1.94 (95% CI -2.00 to -1.87)] (Figure 1A, Table S1). Furthermore, in 2021, ASDR and ASMR for breast cancer attributable to high red meat intake were consistently higher in females than males. Among females, Southern Sub-Saharan Africa exhibited the peak ASMR and ASDR, whereas among males, Eastern Sub-Saharan Africa reported the greatest rates (Figure S2).

**Figure 2:**
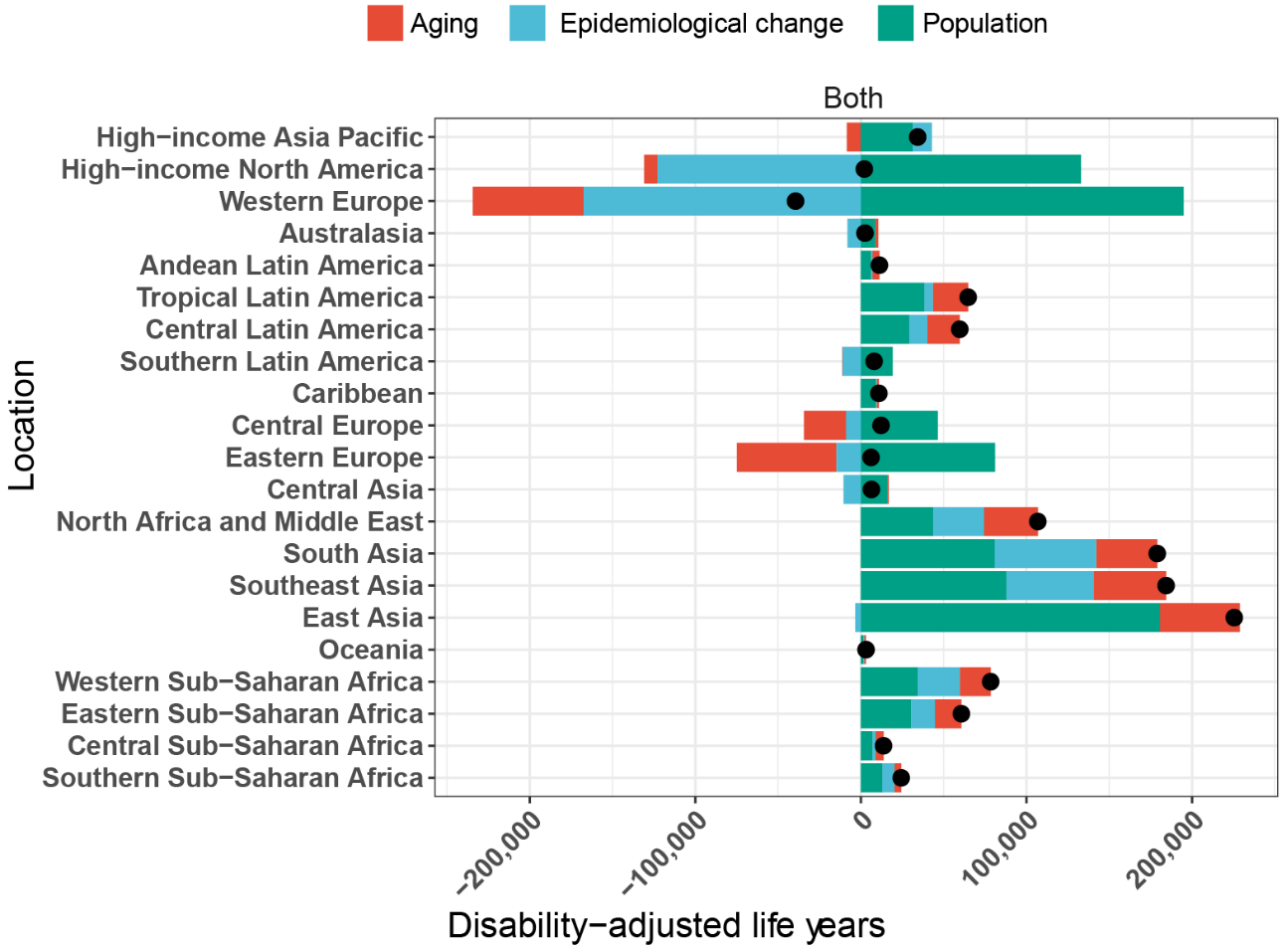
Population-Level Determinant Changes in Aging, Population Growth, and Epidemiological Changes for DALYs of Breast Cancer Attributed to Diet High in Red Meat Across 21 Regions from 1990 to 2021. Black dots represent the total change contributed by all three components. DALYs, disability-adjusted life-years.

 In terms of the decline in DALYs for breast cancer linked to high red meat consumption, aging contributed the most in Eastern Europe (-979.91%), whereas epidemiological changes had the greatest impact in High-income North America (-5849.76%). In terms of DALY expansion, aging had the greatest contribution in Australasia (72.86%), population growth contributed the most in High-income North America (6332.37%), and epidemiological changes had the greatest impact in South Asia (34.22%). Notably, the escalation of DALYs within High-income North America is primarily attributed to population growth, which outweighs the negative effects of aging and epidemiological changes. Although ASMR and ASDR are declining, the increase in population leads to a higher total number of DALYs, explaining the overall DALY increase despite the declining trends in ASRs (Table S1). Overall, population growth was a major contributing factor to the rising disease burden (Figure 2, Table S2).

###  Deaths and DALYs at the National Level

 In 2021, Palau reported the highest ASMR for breast cancer associated with a high-red-meat diet [2.94 per 100 000 (95% UI -0.01–6.51)], while Nauru recorded the greatest ASDR [86.55 per 100 000 (95% UI -0.04–223.99)]. From 1990 to 2021, ASMR showed a declining trend in 68 countries and an increasing trend in 136 countries. Greenland experienced the steepest reduction [EAPC: -2.49 (95% CI -2.66 to -2.33)], while Turkey reported the strongest rise [EAPC: 3.92 (95% CI 3.16 to 4.70)]. Similarly, ASDR decreased in 73 countries and increased in 131 countries. The Kingdom of Denmark demonstrated the greatest downward trend [EAPC: -2.76 (95% CI -2.88 to -2.65)], while Turkey had the highest increase [EAPC: 3.78 (95% CI 3.02 to 4.54)] (Table S3, Figure 1B).

###  Disease Burden Across SDI Strata

 In 2021, the high SDI region recorded the highest ASMR [1.14 per 100 000 (95% UI -0.01–2.43)] and ASDR [33.07 per 100 000 (95% UI -0.02–69.90)] for breast cancer associated with high red meat intake. Between 1990 and 2021, both ASMR and ASDR declined in high-middle and high SDI regions, while the other three SDI regions exhibited a rising trajectory. The sharpest decline was recorded in the high SDI region, whereas the most notable escalation was observed in the low-middle SDI region (Figure 1A, Table S1).

**Figure 3:**
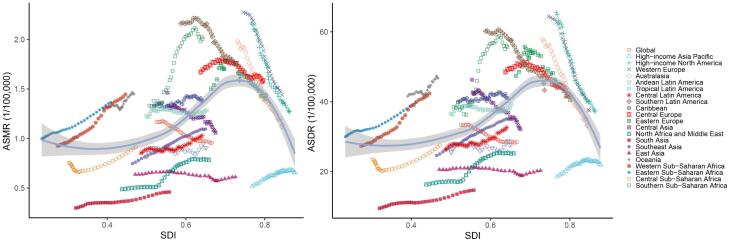
ASRs for Breast Cancer Attributed to Diet High in Red Meat Across 21 Regions by SDI from 1990 to 2021. The solid line represents the overall trend of ASMR and ASDR with changes in SDI, while the shaded grey area indicates the confidence interval, reflecting the variability around this trend. ASRs, Age-standardized rates; ASMR, Age-standardized mortality rate; ASDR, Age-standardized disability-adjusted life-year rate; SDI, Sociodemographic index

**Figure 4:**
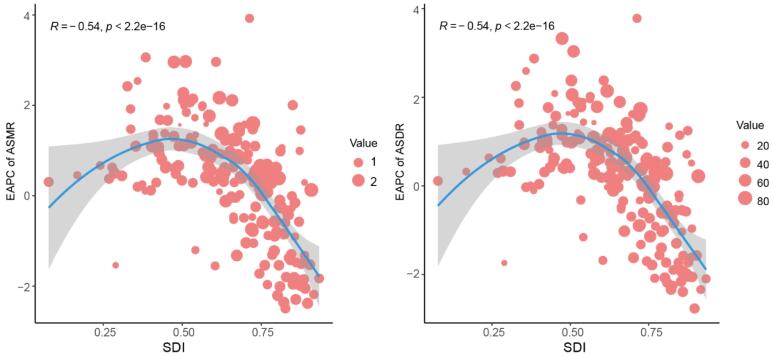
EAPCs of ASRs for Breast Cancer Attributed to Diet High in Red Meat in 204 Countries and Territories by SDI in 2021. The blue line represents the overall trend of EAPCs of ASMR and ASDR with changes in SDI, while the shaded grey area indicates the confidence interval, reflecting the variability around this trend. Each red dot represents the EAPC value of an individual country or region plotted against its SDI, with the size of the dot corresponding to the population size of that country or region, where larger dots represent larger populations. EAPCs, Estimated annual percentage changes; ASRs, Age-standardized rates; ASMR, Age-standardized mortality rate; ASDR, Age-standardized disability-adjusted life-year rate; SDI, Sociodemographic index

 From 1990 to 2021, apart from High-income Asia Pacific, a strong inverse association was identified between ASMR and ASDR of breast cancer attributed to a high-red-meat diet and SDI in high SDI regions, whereas a positive correlation was noted in low- and middle-SDI regions (Figure 3). Additionally, when SDI was below 0.5, the EAPCs of ASMR and ASDR exhibited a direct association with SDI, whereas above 0.5, the trend reversed, and EAPCs tended to become more negative as SDI increased (Figure 4). In 2021, national-level ASMR and ASDR were positively correlated with SDI in low- and middle-SDI regions, but negatively correlated in high SDI regions (Figure S3).

**Figure 5:**
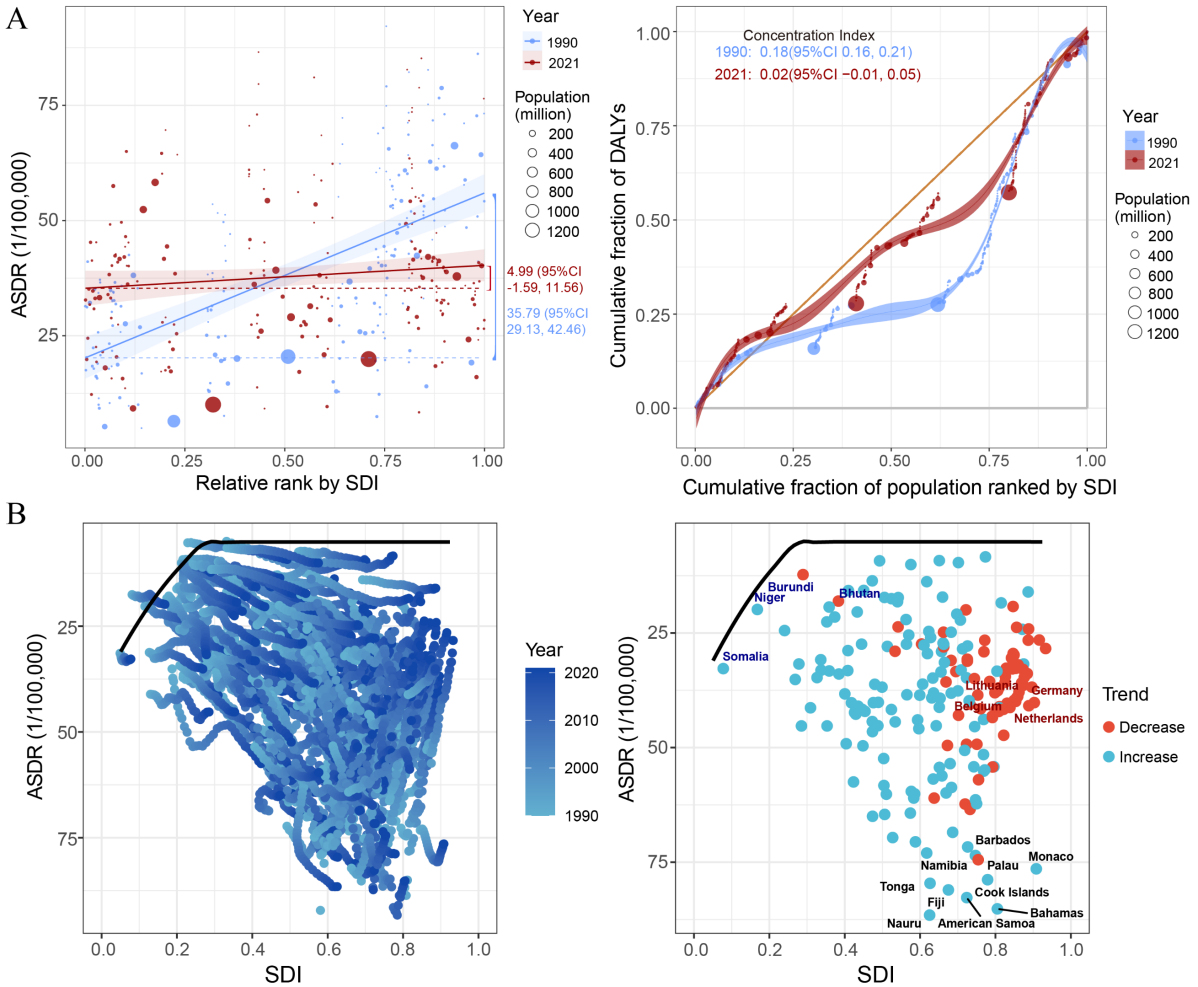
Cross-country Inequality Analysis and Frontier Analysis of the Burden of Breast Cancer Attributed to Diet High in Red Meat. **A.** Health inequality regression curves and concentration curves for DALYs attributed to diet high in red meat in 1990 and 2021, with points representing individual countries and territories sized by population. **B. **Frontier analysis illustrating the relationship between ASDR and SDI for breast cancer attributed to diet high in red meat across 204 countries and territories in 2021. Each point represents a specific country or territory, with the boundary line shown in black. The 10 countries and territories with the greatest differences from the boundary line are marked in black. Blue dots represent low SDI countries and territories with minimal deviation from the frontier, while red dots represent high SDI countries and territories with the largest deviation from the frontier. DALYs, Disability-adjusted life-years; ASDR, Age-standardized DALYs rate; SDI, Sociodemographic index

 Cross-country inequality analysis revealed that the elevated ASDRs of breast cancer linked to a high-red-meat diet were unevenly distributed across nations and regions with elevated SDI levels. The ASDR gap between the top- and bottom-ranked SDI regions narrowed from 35.79 (95% CI 29.13 to 42.46) in 1990 to 4.99 (95% CI -1.59 to 11.56) in 2021. Similarly, the concentration index declined from 0.18 (95% CI 0.16 to 0.21) in 1990 to 0.02 (95% CI -0.01 to 0.05) in 2021, reflecting a substantial reduction in both absolute and relative health inequalities in the burden of breast cancer associated with high red meat consumption over time (Figure 5A).

 Frontier analysis identified the ten leading nations and regions with the greatest potential for ASDR improvement, including Nauru, Bahamas, American Samoa, Fiji, Tonga, Cook Islands, Monaco, Palau, Barbados, and Namibia. Among frontier locations characterized by low SDI, Niger, Burundi, Somalia, and Bhutan were highlighted, while high SDI nations and regions with significant potential for advancement included Lithuania, Belgium, Germany, and the Netherlands (Figure 5B, Table S4).

###  Age- and Sex-Specific Disease Burden

**Figure 6:**
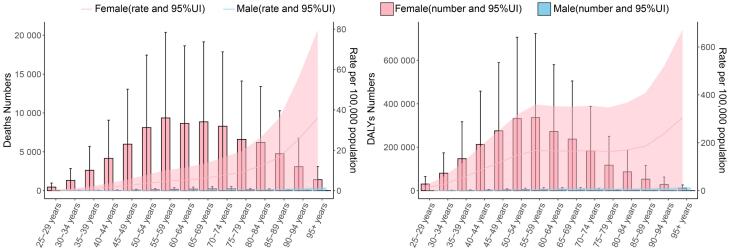
Age-Specific Numbers and Rates of Deaths and DALYs from Breast Cancer Attributable to Diet High in Red Meat by Gender in 2021. The left Y-axis (Deaths/DALYs cases) corresponds to the bars, with blue bars representing males and pink bars representing females (both including 95% uncertainty interval). The right Y-axis (Death/DALYs rate per 100 000 population) corresponds to the line graphs with shaded areas, showing age-specific mortality and DALY rates with 95% uncertainty interval for males (blue line) and females (red line). DALYs, Disability-adjusted life-years

 In 2021, breast cancer DALYs and deaths associated with a high-red-meat diet were predominantly concentrated among females aged 55–59 and males aged 65–69. The age-specific mortality rate increased progressively with advancing years in both sexes. Among women, the age-specific DALY rate remained relatively stable between 55 and 84 years before rising sharply, while in males, a positive correlation between DALY rate and age was consistently observed (Figure 6).

## Discussion

 This study demonstrated that the disease burden of breast cancer linked to a high-red-meat diet changed significantly from 1990 to 2021. In 2021, an estimated 12.07% of breast cancer deaths were associated with a high-red-meat diet, which was among the most prominent diet-related risk factors. However, given the inherent limitations of the GBD dataset, this estimate should be interpreted cautiously rather than as a definitive determinant of breast cancer risk. The potential mechanisms may involve the high saturated fatty acid content in red meat, which can elevate cholesterol and hormone levels, particularly influencing estrogen metabolism.^5^ Additionally, iron derived from red meat could play a role in cellular oxidative imbalance and lipid peroxidation, thereby influencing the development and progression of breast cancer.^4^ It is essential to acknowledge that GBD data is derived from observational studies, and causality cannot be conclusively established. While we discuss mechanisms like heme iron and saturated fat, these should be viewed as potential contributors rather than definitive causes of breast cancer. The observational nature of the data means that other confounding variables—such as processed meat consumption, alcohol intake, and genetic predispositions—may play significant roles, but were not fully captured in this analysis.

 From 1990 to 2021, breast cancer DALYs and deaths associated with a high-red-meat diet increased to varying extents, potentially influenced by demographic expansion, shifts in dietary patterns, and an aging population.^24^ Among these factors, population growth made a particularly significant contribution to the increasing disease burden. Notably, while the ASDR and ASMR for female breast cancer linked to high red meat intake showed a declining trend, those for male breast cancer showed an upward trend. This discrepancy may be explained by a higher proportion of high red meat consumption and elevated obesity rates in men relative to women. Earlier GBD analyses have already found that the proportion of high red meat consumption is generally greater in men than women.^25^ Moreover, male breast cancer remains rare, with limited public and healthcare awareness, and there is currently a lack of targeted screening programs for male breast cancer, potentially resulting in delayed detection and intervention.^2,26^ In 2021, the impact of breast cancer attributed to diet high in red meat was predominantly concentrated among middle-to-older age groups, possibly due to the long-term accumulation of dietary effects, which become more apparent over time and with advancing age.

 However, despite the global decline in ASMR/ASDR, a considerable number of countries (as shown in Table S3) experienced rising ASMR, highlighting notable regional variations. For instance, North Africa and the Middle East exhibited substantial growth in both ASMR and ASDR, highlighting the need for region-specific interventions. This contradiction can be linked to variations in healthcare infrastructure, eating patterns, and risk factors, including the prevalence of red meat in traditional diets and the increasing adoption of Western dietary patterns in rapidly developing regions. As a result, these regions continue to experience a rising disease burden of breast cancer related to excessive red meat intake.

 The impact of breast cancer associated with a high-red-meat diet varies considerably across various nations and geographic regions. Between 1990 and 2021, the ASMR and ASDR of breast cancer linked to excessive red meat consumption exhibited an increasing trend in 13 regions, encompassing tropical, subtropical, and some temperate areas, which are suitable for agricultural and livestock production where red meat is a common component of traditional diets. These regions have undergone significant globalization and urbanization, resulting in a dietary shift from predominantly plant-based diets to high-protein, high-fat Western diets.^27^ With the rapid proliferation of Western fast-food franchises and the increasing influence of convenience-driven eating habits, red meat and processed meat consumption has risen substantially. Additionally, economic development has improved cold chain logistics, making red meat more accessible and affordable, further boosting consumption.^28^ North Africa and the Middle East experienced a particularly significant increase in ASMR from 1990 to 2021, likely due to dietary habits, traditional culture, and economic development. In the sociocultural context of this region, red meat consumption is considered a symbol of wealth and status, and it constitutes a significant portion of the diet. Red meat consumption is particularly prominent during festivals and religious ceremonies.^29^

 As of 2021, among the 10 nations exhibiting the greatest ASMR of breast cancer linked to a high-red-meat diet, 9 were Pacific Island nations, most of which have relatively small populations. Due to geographic isolation, these countries heavily rely on imports for food supply, with red meat playing a significant role.^30^ As globalization accelerated, these nations, often dependent on tourism as an economic pillar, experienced a rapid Westernization of dietary patterns to cater to international tourists. Consequently, red meat became a dietary staple, being cheaper and more readily available compared to fresh fruits and vegetables.^31^ Furthermore, with small population bases, disease data in these countries are more susceptible to being influenced by high-risk factors. The limited scale and resources of local healthcare systems, combined with low coverage of breast cancer screening and early diagnosis, as well as delayed health education and policy interventions targeting dietary habits,^32^ further exacerbate the risks in these countries. However, we must also take into account hereditary predispositions and ecological influences, including pollution and endocrine disruptors, that could play a role in the elevated incidence of breast cancer observed in Pacific Island nations which should be investigated in future studies.

 As of 2021, the high SDI region recorded the greatest ASDR and ASMR associated with breast cancer due to a high-red-meat diet, while also showing the most substantial reduction in ASDR and ASMR over the period from 1990 to 2021. Historically, red meat has served as a staple protein source in high SDI regions, exemplified by beef consumption in North America. With a highly developed food industry, the consumption of convenient, inexpensive, and calorie-dense processed foods has long been prevalent in these regions.^33^ Consequently, the burden of breast cancer due to dietary factors has a long-standing history, with significant cumulative effects that cannot be fully reversed in the short term. However, with the implementation of public health policies (e.g. food labeling regulations, red meat taxation), the promotion of healthy diets and lifestyles,^34^ and advancements in early breast cancer screening and treatment, the disease burden has been effectively mitigated. For example, several economically developed nations have implemented policies to reduce red meat consumption following the International Agency for Research on Cancer’s 2015 classification of processed meats as carcinogenic.^35^ These efforts, along with recommendations from the World Health Organization to reduce red meat intake,^36^ have likely contributed to the decline in breast cancer incidence. Together with improved screening and treatment methods, these policies have successfully reduced both mortality rates and the overall disease burden, underscoring the significance of sustained public health efforts in mitigating breast cancer risk.

 From 1990 to 2021, both absolute and proportional health disparities in breast cancer impact linked to high red meat consumption showed a marked decline. This may be attributed to the accelerated pace of globalization, leading to a gradual Westernization of dietary patterns in low-income nations and regions, with increased red meat consumption and a corresponding rise in health impact. Meanwhile, in middle- and high- income economies, heightened awareness of the health risks associated with red meat intake has prompted residents to reduce their consumption, thereby lowering the public health burden and reducing health inequalities.^37^ Furthermore, some high SDI countries, such as Lithuania, Belgium, Germany, and the Netherlands, have not achieved the expected improvement in reducing the disease burden. This underscores the need for these countries to engage in multidisciplinary collaboration and disease management, with a focus on early intervention, to further alleviate the public health impact of breast cancer attributed to diet high in red meat.

 To mitigate the burden of breast cancer linked to excessive red meat consumption, countries and regions should enhance public awareness of this link and advocate for healthier dietary practices. Additionally, the increasing burden of male breast cancer caused by dietary factors should not be overlooked. Public health policies targeting red meat consumption (e.g. implementing red meat taxes and restrictions on processed foods) should be developed. Low- and middle-income regions and island nations should strengthen early breast cancer screening and intervention,^38^ particularly for middle-aged and elderly populations. Moreover, the experience of high SDI regions in reducing the disease burden provides valuable insights for other regions, demonstrating that dietary optimization and health policy interventions can significantly enhance breast cancer risk mitigation and management.

 This research represents the first comprehensive analysis of the global and regional burden of breast cancer attributed to diet high in red meat. Utilizing the latest epidemiological data from 1990 to 2021, it explores the epidemiological characteristics of this disease across gender, age, countries, regions, and SDI levels. The findings provide critical insights for understanding the SDI-related distribution of the disease and for formulating targeted public health strategies, contributing to the rational allocation and regulation of healthcare resources across regions and fostering a sustainable health-oriented societal environment to mitigate the health impact of breast cancer resulting from a diet high in red meat.

 Nevertheless, this research is subject to certain limitations. First, this evaluation is based on GBD data, which primarily relies on case reports and disease registries collected by various countries and regions. Discrepancies in data acquisition techniques and quality standards across different regions may lead to data omissions and inaccuracies, potentially resulting in an underestimation of the disease burden. Second, due to political and cultural factors in certain regions, GBD data lacks comprehensive coverage of all racial groups and geographic areas, reflecting only the characteristics of specific populations. Third, breast cancer attributable to a diet rich in red meat generally exhibits a time-lag effect, and the impact of risk factor interventions in some countries may not yet be reflected in the disease burden, necessitating long-term observation to assess the effectiveness of these policies. Finally, while red meat has been recognized as a significant dietary determinant, it is only one of many potential contributors to breast cancer, and other dietary and environmental factors should also be considered in future studies.

## Conclusion

 Despite the declining trend in the global impact of breast cancer associated with excessive red meat intake over the period from 1990 to 2021, it remains a significant dietary risk factor with a substantial global impact. However, given the limitations of the GBD dataset, these results should be carefully analyzed, considering the multifactorial nature of breast cancer development. The increasing disease burden in certain regions, driven by population growth and low development levels, should not be overlooked. Therefore, targeted intervention strategies based on gender, age, and location should be implemented to encourage healthier dietary patterns and lifestyle choices, aiming to mitigate the future disease risk linked to high red meat consumption.

## Supplementary Files

Supplementary file 1 contains Tables S1-S4 and Figures S1-S3.
