# Supplementary material for: Burden of Breast Cancer Attributable to a Diet High in Red Meat at Global, Regional, and National Levels: An Analysis of the Global Burden of Disease Study 2021
Source: Arch Iran Med. 2025 May 1;28(5):275–85. doi: 10.34172/aim.34079 (PMC12305419; doi:10.34172/aim.34079)
Supplement: Supplementary file 1 — contains Tables S1-S4 and Figures S1-S3. [file aim-28-275-s001.pdf]

Arch Iran Med. May 2025;28(5):275-S1
10.34172/aim.34079
http://journalaim.com

Table S1: Deaths and disability-adjusted life years due to breast cancer attributed to diet high in red meat across 21 regions and 5 sociodemographic index regions from 1990 to 2021.

Location	1990				2021				1990-2021	
	**Deaths (95% UI)**	**DALYs (95% UI)**	**ASMR/100,000 persons (95% UI)**	**ASDR/100,000 persons (95% UI)**	**Deaths (95% UI)**	**DALYs (95% UI)**	**ASMR/100,000 persons (95% UI)**	**ASDR/100,000 persons (95% UI)**	**EAPC of ASMR (95% CI)**	**EAPC of ASDR (95% CI)**
Andean Latin America	179.57(-0.08-388.58)	6125.41(-3.06-13296.82)	0.82(-0.00-1.77)	25.57(-0.01-55.46)	549.24(-0.18-1209.93)	17336.73(-5.90-37815.12)	0.91(-0.00-2.01)	27.65(-0.01-60.10)	0.12(0.00-0.24)	0.04(-0.08-0.16)
Australasia	449.43(-0.27-960.23)	12991.63(-8.97-27907.90)	1.98(-0.00-4.24)	57.73(-0.04-124.06)	597.38(-0.43-1268.79)	15501.92(-9.37-32639.79)	1.14(-0.00-2.41)	33.40(-0.02-70.76)	-1.85(-1.92--1.79)	-1.84(-1.90--1.79)
Caribbean	344.80(-0.17-733.73)	10917.04(-5.69-23406.48)	1.33(-0.00-2.83)	40.02(-0.02-85.65)	743.20(-0.27-1632.58)	21794.96(-8.25-47932.67)	1.38(-0.00-3.04)	41.05(-0.02-90.28)	0.22(0.16-0.29)	0.17(0.11-0.22)
Central Asia	703.11(-0.33-1482.82)	23456.66(-11.13-49808.66)	1.46(-0.00-3.09)	46.29(-0.02-98.31)	905.00(-0.44-1967.52)	29845.58(-14.76-64385.92)	1.06(-0.00-2.31)	32.11(-0.02-69.57)	-0.74(-0.83--0.66)	-1.00(-1.08--0.92)
Central Europe	2409.26(-0.82-5130.19)	71105.66(-23.94-150331.36)	1.67(-0.00-3.54)	48.52(-0.02-102.63)	3443.09(-1.48-7326.62)	83271.45(-36.17-177888.41)	1.58(-0.00-3.37)	42.10(-0.02-90.21)	-0.35(-0.45--0.24)	-0.61(-0.72--0.50)

Central Latin America	757.33(-0.27-1616.57)	26160.40(-8.83-55615.58)	0.85(-0.00-1.82)	26.27(-0.01-55.65)	2632.16(-1.13-5619.09)	85891.40(-35.99-182264.39)	1.03(-0.00-2.20)	32.62(-0.01-69.31)	0.48(0.38-0.59)	0.54(0.44-0.64)
Central Sub-Saharan Africa	186.61(-0.02-418.17)	6674.97(-0.88-15040.02)	0.75(-0.00-1.70)	23.45(-0.00-52.36)	564.21(-0.13-1324.53)	20381.60(-4.46-48618.87)	0.91(-0.00-2.14)	27.44(-0.01-64.56)	0.69(0.41-0.98)	0.59(0.30-0.88)
East Asia	5809.64(-1.75-12737.52)	209989.85(-67.05-462189.23)	0.64(-0.00-1.40)	20.57(-0.01-45.30)	13178.61(-6.73-28699.29)	435514.30(-244.71-958943.16)	0.61(-0.00-1.33)	20.25(-0.01-44.59)	-0.40(-0.52--0.28)	-0.27(-0.37--0.17)
Eastern Europe	4104.86(-1.33-8723.38)	129042.63(-39.07-275785.05)	1.50(-0.00-3.20)	47.41(-0.01-101.32)	4928.16(-1.96-10446.37)	135182.85(-52.43-289880.01)	1.44(-0.00-3.05)	41.51(-0.02-89.37)	-0.56(-0.79--0.32)	-0.89(-1.12--0.65)
Eastern Sub-Saharan Africa	791.22(-0.12-1776.49)	27826.51(-4.62-62404.46)	1.00(-0.00-2.25)	29.96(-0.00-67.39)	2503.27(-0.60-5616.96)	88540.53(-23.64-198001.35)	1.37(-0.00-3.04)	39.62(-0.01-88.37)	1.01(0.91-1.11)	0.85(0.75-0.95)
Global	45073.85(-13.31-96485.06)	1396840.46(-435.82-3004079.75)	1.17(-0.00-2.50)	33.31(-0.01-71.68)	81506.23(-25.57-175444.92)	2451718.64(-790.88-5232217.29)	0.96(-0.00-2.06)	28.37(-0.01-60.54)	-0.77(-0.82--0.72)	-0.65(-0.70--0.60)
High-income Asia Pacific	1052.76(-0.42-2227.02)	38343.13(-16.36-81540.48)	0.52(-0.00-1.09)	18.52(-0.01-39.35)	2684.73(-0.76-5799.42)	72649.84(-23.35-156826.71)	0.65(-0.00-1.41)	21.91(-0.01-46.74)	0.77(0.62-0.91)	0.53(0.36-0.69)

High-income North America	7382.48(-3.15-15769.40)	212999.85(-96.23-454542.64)	2.17(-0.00-4.63)	65.49(-0.03-139.71)	8147.48(-3.66-17523.08)	215098.79(-98.81-461979.22)	1.27(-0.00-2.73)	37.35(-0.02-80.25)	-1.86(-1.92--1.79)	-1.94(-2.00--1.87)
High-middle SDI	12813.26(-4.30-27374.34)	400673.84(-141.12-849145.61)	1.32(-0.00-2.82)	39.07(-0.01-82.90)	20004.84(-8.22-42658.17)	573498.85(-245.35-1202058.93)	1.03(-0.00-2.21)	30.26(-0.01-63.41)	-0.95(-1.06--0.84)	-1.01(-1.10--0.92)
High SDI	19775.11(-6.78-42142.06)	554199.38(-221.21-1177795.37)	1.83(-0.00-3.91)	52.82(-0.02-112.23)	23594.03(-9.08-50667.42)	595239.55(-263.94-1258102.87)	1.14(-0.00-2.43)	33.07(-0.02-69.90)	-1.63(-1.67--1.58)	-1.59(-1.63--1.55)
Low-middle SDI	3087.26(-0.70-6710.73)	109939.74(-25.34-237736.42)	0.47(-0.00-1.01)	14.81(-0.00-32.23)	11451.25(-2.91-24934.29)	396905.97(-108.34-865030.67)	0.75(-0.00-1.63)	23.99(-0.01-52.28)	1.55(1.52-1.58)	1.56(1.52-1.59)
Low SDI	1613.19(-0.33-3556.80)	56861.29(-13.22-125498.74)	0.67(-0.00-1.46)	20.54(-0.00-45.23)	4850.52(-1.17-10506.92)	172752.21(-44.96-372552.44)	0.87(-0.00-1.90)	26.62(-0.01-57.42)	0.85(0.72-0.98)	0.78(0.65-0.90)
Middle SDI	7717.36(-2.14-16753.04)	273123.51(-76.77-596910.65)	0.71(-0.00-1.54)	22.11(-0.01-48.11)	21496.61(-5.77-46626.15)	710319.34(-197.87-1524947.64)	0.79(-0.00-1.71)	24.95(-0.01-53.63)	0.21(0.16-0.26)	0.26(0.21-0.32)
North Africa and Middle East	914.52(-0.33-1948.89)	33894.72(-13.62-72970.30)	0.49(-0.00-1.05)	16.32(-0.01-34.90)	3901.04(-1.27-8370.36)	140767.40(-46.76-300790.68)	0.79(-0.00-1.69)	25.24(-0.01-53.95)	2.03(1.79-2.26)	1.83(1.62-2.03)

Oceania	43.16(-0.01-93.70)	1634.56(-0.55-3560.65)	1.30(-0.00-2.81)	41.53(-0.01-90.41)	127.00(-0.04-279.19)	4770.62(-1.45-10505.23)	1.45(-0.00-3.20)	46.72(-0.01-102.98)	0.34(0.25-0.43)	0.36(0.27-0.46)
South Asia	1897.72(-0.23-4156.49)	67949.24(-8.15-148571.99)	0.30(-0.00-0.66)	9.48(-0.00-20.74)	7157.56(-0.83-15824.04)	246938.78(-31.53-546997.17)	0.46(-0.00-1.02)	14.67(-0.00-32.37)	1.22(1.08-1.36)	1.24(1.09-1.38)
Southeast Asia	2125.17(-0.38-4688.44)	77791.64(-14.53-172198.55)	0.75(-0.00-1.64)	24.58(-0.00-54.21)	7646.64(-1.96-17187.96)	262141.64(-67.85-590574.64)	1.10(-0.00-2.45)	35.05(-0.01-78.80)	1.22(1.16-1.28)	1.12(1.06-1.18)
Southern Latin America	979.05(-0.72-2085.45)	27957.95(-16.79-59703.90)	2.16(-0.00-4.61)	60.16(-0.04-128.38)	1378.68(-0.84-2983.97)	35964.92(-24.42-76954.88)	1.59(-0.00-3.44)	43.29(-0.03-92.42)	-0.93(-1.07--0.80)	-1.02(-1.11--0.92)
Southern Sub-Saharan Africa	368.34(-0.14-819.91)	12262.66(-4.93-27092.67)	1.33(-0.00-2.94)	38.57(-0.02-85.42)	1153.66(-0.37-2508.12)	36635.00(-13.73-80377.36)	2.01(-0.00-4.38)	55.65(-0.02-121.23)	1.74(1.48-1.99)	1.74(1.45-2.03)
Tropical Latin America	1161.83(-0.43-2472.76)	38685.10(-14.79-81873.94)	1.23(-0.00-2.61)	36.53(-0.01-77.44)	3336.36(-1.96-7156.61)	103494.43(-58.69-220712.21)	1.29(-0.00-2.76)	39.19(-0.02-83.61)	-0.02(-0.09-0.05)	-0.01(-0.08-0.06)
Western Europe	12566.38(-4.08-26818.23)	332829.04(-124.60-702493.95)	2.27(-0.00-4.84)	64.42(-0.03-135.84)	12823.73(-4.97-27476.29)	293387.63(-132.68-630172.01)	1.36(-0.00-2.90)	37.42(-0.02-80.12)	-1.74(-1.79--1.68)	-1.81(-1.87--1.76)

Western Sub-Saharan Africa	846.62(-0.17-1772.46)	28201.80(-6.41-59300.98)	0.93(-0.00-1.94)	27.26(-0.01-57.25)	3105.04(-0.65-6794.11)	106608.27(-24.84-235377.15)	1.44(-0.00-3.14)	42.35(-0.01-92.52)	1.59(1.45-1.73)	1.58(1.43-1.72)

Table S2: Age-standardized disability-adjusted life year rates of breast cancer attributed to diet high in red meat with decomposition analysis, categorized by 21 regions.

Location	Overll difference	Aging	Population	Epidemiological change	Percent change of aging	Percent change of population	Percent change of epidemiological change
Southeast Asia	184350	43885.32	88112.06	52352.62	23.81	47.8	28.4
East Asia	225524.44	48386.17	180522.24	-3383.97	21.45	80.05	-1.5
Oceania	3136.06	1061.88	1707.74	366.44	33.86	54.45	11.68
Central Asia	6388.91	749.57	16116.51	-10477.17	11.73	252.26	-163.99
Eastern Europe	6140.22	-60168.68	81009.92	-14701.02	-979.91	1319.33	-239.42
Central Europe	12165.79	-25393.74	46473.59	-8914.07	-208.73	382	-73.27
High-income Asia Pacific	34306.72	-8504.23	31271.11	11539.83	-24.79	91.15	33.64
Western Europe	-39441.41	-67000.19	194933.51	-167374.74	169.87	-494.24	424.36
Australasia	2510.29	1829.02	8742.57	-8061.3	72.86	348.27	-321.13
High-income North America	2098.94	-8030.72	132912.49	-122782.84	-382.61	6332.37	-5849.76
Southern Latin America	8006.97	-331.47	19205.6	-10867.15	-4.14	239.86	-135.72
Caribbean	10877.92	1246.84	9237.19	393.89	11.46	84.92	3.62
Andean Latin America	11211.31	4207.57	6283.15	720.59	37.53	56.04	6.43
Central Latin America	59731	19630.93	29188.97	10911.09	32.87	48.87	18.27
North Africa and Middle East	106872.67	32553.32	43788.53	30530.82	30.46	40.97	28.57
Tropical Latin America	64809.33	21389.12	38489.13	4931.07	33	59.39	7.61
South Asia	178989.54	36877.65	80862.96	61248.93	20.6	45.18	34.22
Eastern Sub-Saharan Africa	60714.01	16040.73	30507.04	14166.24	26.42	50.25	23.33

Central Sub-Saharan Africa	13706.64	4780.89	7160.78	1764.96	34.88	52.24	12.88
Southern Sub-Saharan Africa	24372.34	4043.04	12944.76	7384.55	16.59	53.11	30.3
Western Sub-Saharan Africa	78406.47	18704.95	34363.22	25338.3	23.86	43.83	32.32

Table S3: Deaths and disability-adjusted life years due to breast cancer attributed to diet high in red meat across 204 countries and territories from 1990 to 2021.

**Location**	**1990**				**2021**				**1990-2021**	
	**Deaths (95% UI)**	**DALYs (95% UI)**	**ASMR/100,000 persons (95% UI)**	**ASDR/100,000 persons (95% UI)**	**Deaths (95% UI)**	**DALYs (95% UI)**	**ASMR/100,000 persons (95% UI)**	**ASDR/100,000 persons (95% UI)**	**EAPC of ASMR (95% CI)**	**EAPC of ASDR (95% CI)**
American Samoa	0.43(-0.00-0.93)	14.90(-0.01-33.56)	1.90(-0.00-4.16)	53.29(-0.02-115.48)	1.34(-0.00-2.97)	42.71(-0.02-95.38)	2.83(-0.00-6.26)	82.73(-0.03-186.53)	1.60(1.49-1.72)	1.73(1.62-1.85)
Antigua and Barbuda	1.04(-0.00-2.22)	28.58(-0.01-60.86)	1.97(-0.00-4.21)	56.69(-0.02-121.10)	2.37(-0.00-5.03)	69.51(-0.02-146.12)	2.25(-0.00-4.78)	62.25(-0.02-130.90)	0.64(0.43-0.85)	0.54(0.33-0.74)
Arab Republic of Egypt	184.17(-0.06-392.57)	7212.04(-2.70-15375.27)	0.57(-0.00-1.20)	19.56(-0.01-41.78)	808.37(-0.40-1807.61)	28644.70(-15.50-63898.28)	1.17(-0.00-2.60)	36.15(-0.02-80.67)	2.96(2.51-3.41)	2.38(2.01-2.74)
Argentine Republic	742.39(-0.53-1590.24)	21085.56(-10.07-45106.16)	2.35(-0.00-5.03)	65.56(-0.03-140.20)	1007.61(-0.68-2171.11)	26427.16(-20.08-56783.00)	1.81(-0.00-3.90)	49.28(-0.04-105.99)	-0.78(-0.94--0.61)	-0.88(-1.02--0.74)
Australia	357.41(-0.22-761.59)	10286.69(-6.97-21970.45)	1.89(-0.00-4.03)	54.72(-0.04-116.97)	493.12(-0.35-1053.65)	12608.46(-7.38-26828.62)	1.11(-0.00-2.36)	32.17(-0.02-68.66)	-1.83(-1.90--1.75)	-1.84(-1.90--1.77)
Barbados	6.70(-0.01-14.24)	180.41(-0.13-386.33)	2.46(-0.00-5.23)	71.23(-0.05-152.12)	13.15(-0.01-29.86)	342.75(-0.21-773.50)	2.65(-0.00-6.02)	73.56(-0.05-166.40)	0.66(0.44-0.89)	0.52(0.33-0.71)

Belize	0.62(-0.00-1.35)	19.54(-0.01-42.19)	0.66(-0.00-1.42)	19.70(-0.01-42.59)	2.87(-0.00-6.31)	98.67(-0.03-214.93)	0.88(-0.00-1.95)	27.66(-0.01-60.39)	0.94(0.62-1.27)	1.08(0.79-1.37)
Bermuda	1.73(-0.00-3.66)	48.35(-0.01-101.43)	2.85(-0.00-6.04)	75.21(-0.02-158.09)	2.08(-0.00-4.59)	49.07(-0.03-108.67)	1.53(-0.00-3.37)	41.65(-0.02-92.34)	-2.36(-2.58--2.14)	-2.34(-2.57--2.10)
Bolivarian Republic of Venezuela	107.18(-0.08-228.35)	3774.13(-2.69-8012.13)	1.02(-0.00-2.16)	32.06(-0.02-67.84)	444.11(-0.26-982.67)	14305.33(-7.68-32134.41)	1.46(-0.00-3.23)	45.87(-0.02-102.71)	1.03(0.87-1.19)	0.97(0.80-1.14)
Bosnia and Herzegovina	40.46(-0.01-89.82)	1357.09(-0.32-2987.45)	0.95(-0.00-2.14)	29.65(-0.01-65.05)	77.16(-0.03-172.23)	1978.46(-0.94-4486.23)	1.27(-0.00-2.84)	34.77(-0.02-79.37)	1.47(1.28-1.66)	1.01(0.81-1.21)
Brunei Darussalam	1.47(-0.00-3.34)	58.07(-0.03-133.53)	1.12(-0.00-2.49)	37.53(-0.02-84.28)	5.21(-0.00-11.88)	194.47(-0.11-443.39)	1.26(-0.00-2.89)	41.31(-0.02-93.64)	0.98(0.76-1.21)	0.87(0.63-1.12)
Burkina Faso	59.59(-0.01-129.31)	2054.78(-0.61-4535.75)	1.37(-0.00-2.94)	41.08(-0.01-90.05)	153.78(-0.08-354.72)	5277.16(-2.81-12427.87)	1.59(-0.00-3.63)	45.28(-0.02-105.34)	0.50(0.40-0.60)	0.33(0.22-0.45)
Canada	658.86(-0.32-1406.16)	18531.65(-8.89-39744.86)	2.06(-0.00-4.40)	58.81(-0.03-126.07)	837.17(-0.43-1801.02)	20492.38(-10.29-44303.60)	1.17(-0.00-2.53)	32.95(-0.02-71.75)	-1.94(-2.01--1.86)	-1.98(-2.05--1.91)
Central African Republic	14.86(-0.00-33.89)	529.34(-0.12-1211.44)	1.21(-0.00-2.75)	36.57(-0.01-82.85)	34.48(-0.01-80.62)	1257.48(-0.53-2959.88)	1.41(-0.00-3.21)	41.32(-0.02-96.68)	0.44(0.39-0.49)	0.32(0.26-0.38)
Commonwealth of Dominica	1.29(-0.00-2.81)	34.17(-0.01-73.66)	2.25(-0.00-4.86)	60.63(-0.01-130.92)	1.86(-0.00-4.15)	50.36(-0.04-113.16)	2.31(-0.00-5.17)	61.40(-0.04-136.93)	0.18(0.10-0.25)	0.10(0.02-0.19)
Commonwealth of the Bahamas	4.39(-0.00-9.34)	151.13(-0.02-324.24)	2.66(-0.00-5.64)	82.69(-0.01-177.31)	11.64(-0.01-25.40)	377.28(-0.21-830.33)	2.81(-0.00-6.16)	85.19(-0.05-187.15)	0.39(0.25-0.53)	0.28(0.15-0.41)

Cook Islands	0.32(-0.00-0.71)	10.37(-0.00-22.97)	2.65(-0.00-5.76)	74.87(-0.03-165.32)	0.69(-0.00-1.52)	18.46(-0.01-41.56)	2.81(-0.00-6.23)	78.83(-0.04-180.22)	0.30(0.15-0.46)	0.33(0.13-0.52)
Czech Republic	266.52(-0.02-575.60)	7003.64(-0.30-14960.49)	1.98(-0.00-4.27)	53.09(-0.00-113.38)	270.73(-0.14-593.90)	6245.15(-2.65-13754.83)	1.26(-0.00-2.78)	32.68(-0.01-71.86)	-1.80(-2.00--1.59)	-1.90(-2.07--1.72)
Democratic People's Republic of Korea	127.44(-0.02-306.33)	4556.57(-1.02-10985.56)	0.72(-0.00-1.69)	23.66(-0.01-56.15)	259.11(-0.06-619.60)	8777.01(-2.29-21378.65)	0.77(-0.00-1.83)	25.44(-0.01-61.56)	0.42(0.32-0.53)	0.45(0.36-0.55)
Democratic Republic of Sao Tome and Principe	0.21(-0.00-0.47)	6.54(-0.00-15.08)	0.33(-0.00-0.74)	9.79(-0.00-22.43)	0.71(-0.00-1.63)	25.24(-0.00-60.10)	0.56(-0.00-1.29)	17.24(-0.00-39.94)	1.98(1.83-2.13)	1.99(1.87-2.11)
Democratic Republic of the Congo	103.28(-0.01-236.04)	3718.51(-0.25-8496.98)	0.59(-0.00-1.35)	18.73(-0.00-43.05)	246.78(-0.02-599.51)	8853.57(-0.54-21943.11)	0.60(-0.00-1.45)	18.04(-0.00-43.79)	0.11(-0.41-0.63)	-0.10(-0.65-0.45)
Democratic Republic of Timor-Leste	2.01(-0.00-4.83)	75.04(-0.01-181.02)	0.59(-0.00-1.37)	18.37(-0.00-43.99)	7.66(-0.00-17.73)	255.55(-0.08-587.50)	0.87(-0.00-2.01)	27.43(-0.01-63.34)	1.38(1.17-1.59)	1.37(1.13-1.62)
Democratic Socialist Republic of Sri Lanka	27.59(-0.00-60.57)	982.40(-0.07-2188.98)	0.24(-0.00-0.52)	7.47(-0.00-16.47)	83.56(-0.01-209.55)	2595.92(-0.15-6447.48)	0.31(-0.00-0.78)	9.64(-0.00-23.73)	1.09(1.00-1.19)	1.01(0.91-1.12)
Dominican Republic	31.62(-0.01-69.77)	1094.73(-0.26-2426.38)	0.83(-0.00-1.82)	24.95(-0.01-55.09)	100.78(-0.06-224.62)	3127.28(-1.88-6995.23)	0.99(-0.00-2.19)	29.50(-0.02-65.83)	0.94(0.72-1.16)	0.86(0.68-1.04)
Eastern Republic of Uruguay	103.44(-0.05-220.93)	2852.52(-1.82-6070.87)	2.76(-0.00-5.87)	79.11(-0.06-167.91)	127.89(-0.06-277.04)	3028.00(-1.60-6455.67)	2.31(-0.00-4.99)	62.32(-0.04-133.23)	-0.74(-0.84--0.64)	-0.89(-0.98--0.81)

Federal Democratic Republic of Ethiopia	224.04(-0.02-580.11)	8085.53(-0.90-21383.36)	1.07(-0.00-2.66)	32.15(-0.00-83.26)	548.67(-0.09-1209.63)	18981.16(-3.13-42089.82)	1.17(-0.00-2.58)	33.28(-0.01-73.76)	0.20(0.01-0.39)	-0.03(-0.20-0.14)
Federal Democratic Republic of Nepal	52.56(-0.01-113.58)	1939.03(-0.38-4214.26)	0.49(-0.00-1.08)	16.19(-0.00-34.93)	153.93(-0.04-344.43)	5335.46(-1.35-11995.84)	0.64(-0.00-1.42)	20.41(-0.00-45.69)	0.89(0.61-1.18)	0.81(0.51-1.10)
Federal Republic of Germany	2831.39(-0.60-6111.62)	73958.16(-16.27-157279.36)	2.30(-0.00-4.97)	64.30(-0.02-136.47)	2855.81(-1.54-6132.43)	64682.10(-33.99-138544.03)	1.47(-0.00-3.13)	40.18(-0.02-85.80)	-1.52(-1.63--1.42)	-1.57(-1.66--1.48)
Federal Republic of Nigeria	437.49(-0.04-967.15)	13943.73(-1.54-30884.46)	0.97(-0.00-2.13)	27.61(-0.00-60.95)	1804.80(-0.20-4141.16)	61083.79(-7.23-141050.59)	1.80(-0.00-4.05)	52.39(-0.01-120.72)	2.27(2.04-2.50)	2.38(2.15-2.61)
Federal Republic of Somalia	29.43(-0.02-70.42)	1097.95(-0.67-2582.06)	1.06(-0.00-2.55)	31.41(-0.02-75.40)	77.08(-0.04-179.78)	2813.72(-1.29-6573.24)	1.15(-0.00-2.69)	32.77(-0.02-76.85)	0.31(0.26-0.36)	0.11(0.06-0.16)
Federated States of Micronesia	0.90(-0.00-2.05)	30.66(-0.01-70.96)	1.83(-0.00-4.14)	55.15(-0.03-126.26)	1.80(-0.00-4.23)	61.78(-0.03-147.35)	2.38(-0.00-5.57)	70.56(-0.03-165.09)	0.86(0.80-0.92)	0.79(0.73-0.85)
Federative Republic of Brazil	1140.90(-0.43-2425.63)	38001.23(-14.79-80378.79)	1.23(-0.00-2.62)	36.76(-0.01-77.86)	3258.71(-1.94-6980.64)	101087.97(-58.07-215098.07)	1.29(-0.00-2.76)	39.23(-0.02-83.51)	-0.05(-0.12-0.02)	-0.03(-0.10-0.04)
French Republic	1775.12(-1.06-3753.06)	45339.28(-36.20-95178.68)	2.22(-0.00-4.68)	61.28(-0.04-128.91)	2104.37(-0.74-4500.93)	47671.84(-21.47-102971.98)	1.45(-0.00-3.12)	41.25(-0.02-89.17)	-1.37(-1.49--1.26)	-1.30(-1.43--1.16)

Gabonese Republic	9.08(-0.00-20.75)	284.74(-0.03-653.32)	1.62(-0.00-3.65)	47.07(-0.01-107.91)	21.51(-0.01-49.88)	717.44(-0.45-1701.82)	2.01(-0.00-4.61)	56.86(-0.03-132.40)	0.53(0.41-0.64)	0.42(0.30-0.54)
Georgia	130.80(-0.10-282.35)	4219.80(-3.13-9087.71)	2.13(-0.00-4.58)	68.64(-0.05-147.73)	128.36(-0.06-281.18)	3434.34(-1.73-7459.50)	2.22(-0.00-4.85)	63.48(-0.03-137.64)	0.55(0.23-0.86)	0.02(-0.24-0.27)
Grand Duchy of Luxembourg	13.63(-0.01-29.16)	362.99(-0.19-784.06)	2.59(-0.00-5.55)	71.09(-0.04-153.81)	14.28(-0.01-31.06)	340.55(-0.18-738.62)	1.30(-0.00-2.81)	33.86(-0.02-73.61)	-1.85(-2.01--1.69)	-2.06(-2.23--1.89)
Greenland	0.79(-0.00-1.74)	26.81(-0.02-58.92)	2.27(-0.00-4.95)	63.42(-0.04-138.91)	0.78(-0.00-1.74)	25.40(-0.01-57.79)	1.14(-0.00-2.54)	34.46(-0.01-77.17)	-2.49(-2.66--2.33)	-2.25(-2.39--2.11)
Grenada	1.12(-0.00-2.43)	32.47(-0.00-70.44)	1.65(-0.00-3.59)	51.26(-0.00-111.18)	2.34(-0.00-5.14)	70.05(-0.01-152.06)	2.15(-0.00-4.72)	59.90(-0.01-130.02)	1.23(0.97-1.49)	0.80(0.55-1.05)
Guam	0.90(-0.00-1.91)	29.53(-0.01-61.94)	1.32(-0.00-2.77)	33.09(-0.01-70.59)	1.93(-0.00-4.29)	64.60(-0.03-144.11)	0.96(-0.00-2.11)	33.33(-0.01-73.41)	-0.26(-0.58-0.05)	0.54(0.33-0.74)
Hashemite Kingdom of Jordan	14.96(-0.00-33.49)	560.86(-0.18-1281.27)	0.95(-0.00-2.09)	31.19(-0.01-70.09)	82.78(-0.02-186.28)	3021.24(-0.94-6829.08)	0.99(-0.00-2.25)	31.14(-0.01-70.14)	0.29(-0.07-0.65)	0.07(-0.31-0.45)
Hellenic Republic	273.31(-0.02-582.22)	7711.19(-0.39-16365.21)	1.90(-0.00-4.03)	55.46(-0.00-117.70)	397.92(-0.27-848.73)	8620.45(-6.51-18233.47)	1.61(-0.00-3.41)	43.44(-0.04-91.89)	-0.89(-1.08--0.70)	-1.03(-1.16--0.90)
Hungary	294.60(-0.11-621.43)	8267.86(-2.68-17492.96)	2.11(-0.00-4.46)	60.16(-0.02-127.91)	304.33(-0.16-659.42)	7391.24(-4.73-15979.30)	1.59(-0.00-3.44)	43.19(-0.03-93.71)	-1.22(-1.38--1.06)	-1.40(-1.55--1.25)
Independent State of Papua New Guinea	22.76(-0.01-52.03)	895.44(-0.38-2003.99)	1.01(-0.00-2.30)	35.30(-0.01-80.49)	77.07(-0.03-178.19)	3076.94(-1.32-7169.07)	1.14(-0.00-2.64)	40.23(-0.02-93.27)	0.29(0.18-0.40)	0.31(0.19-0.43)

Independent State of Samoa	1.05(-0.00-2.32)	33.96(-0.01-75.10)	1.24(-0.00-2.73)	36.26(-0.01-80.22)	2.18(-0.00-4.99)	70.40(-0.03-163.92)	1.47(-0.00-3.36)	43.96(-0.02-101.61)	0.55(0.48-0.62)	0.61(0.53-0.69)
Ireland	96.36(-0.05-202.88)	2684.40(-1.48-5674.55)	2.51(-0.00-5.29)	71.73(-0.04-151.40)	98.78(-0.04-210.82)	2632.74(-1.13-5682.00)	1.27(-0.00-2.71)	36.42(-0.02-78.64)	-1.99(-2.10--1.89)	-2.05(-2.15--1.94)
Islamic Republic of Afghanistan	46.08(-0.02-135.12)	1628.70(-0.61-4938.46)	0.65(-0.00-1.86)	22.38(-0.01-67.62)	127.06(-0.04-369.34)	5051.98(-1.63-14958.54)	0.98(-0.00-2.63)	33.12(-0.01-94.08)	1.47(1.38-1.57)	1.37(1.29-1.46)
Islamic Republic of Iran	121.98(-0.03-264.32)	4788.60(-1.53-10285.35)	0.42(-0.00-0.92)	14.64(-0.00-31.55)	515.89(-0.11-1101.17)	19738.66(-4.14-42148.33)	0.60(-0.00-1.28)	20.89(-0.00-44.79)	1.58(1.28-1.88)	1.62(1.31-1.93)
Islamic Republic of Mauritania	10.77(-0.01-24.36)	336.67(-0.28-778.10)	1.07(-0.00-2.39)	30.77(-0.03-70.50)	28.35(-0.01-62.67)	924.44(-0.53-2096.01)	1.26(-0.00-2.75)	36.81(-0.02-82.20)	0.34(0.21-0.47)	0.40(0.28-0.53)
Islamic Republic of Pakistan	717.49(-0.21-1625.40)	24961.15(-7.61-56750.67)	1.19(-0.00-2.71)	38.11(-0.01-86.57)	2588.67(-0.74-5723.88)	93775.57(-28.26-209080.77)	1.87(-0.00-4.14)	58.28(-0.02-128.75)	1.12(0.91-1.33)	0.98(0.76-1.20)
Jamaica	24.92(-0.01-52.62)	708.85(-0.20-1512.81)	1.44(-0.00-3.03)	42.06(-0.01-89.92)	59.74(-0.02-131.50)	1808.37(-0.55-4056.59)	1.90(-0.00-4.19)	58.70(-0.02-131.63)	0.82(0.51-1.14)	1.03(0.70-1.35)
Japan	886.30(-0.28-1875.65)	31825.43(-10.94-66922.33)	0.53(-0.00-1.12)	19.18(-0.01-40.36)	2223.94(-0.59-4835.72)	57065.65(-15.61-123981.01)	0.72(-0.00-1.57)	24.19(-0.01-51.64)	0.99(0.84-1.14)	0.71(0.53-0.89)
Kingdom of Bahrain	3.00(-0.00-6.51)	110.69(-0.04-241.12)	1.54(-0.00-3.37)	43.25(-0.01-94.17)	13.32(-0.01-30.02)	492.25(-0.19-1097.55)	1.42(-0.00-3.22)	38.54(-0.02-86.23)	-0.56(-0.78--0.33)	-0.73(-0.94--0.52)

Kingdom of Belgium	401.81(-0.30-851.21)	10508.95(-8.24-22177.07)	2.74(-0.00-5.80)	76.59(-0.06-161.87)	354.01(-0.18-767.71)	8028.12(-5.27-17250.64)	1.48(-0.00-3.22)	39.92(-0.03-86.17)	-1.95(-2.05--1.85)	-2.15(-2.31--2.00)
Kingdom of Bhutan	0.99(-0.00-2.28)	37.36(-0.01-86.51)	0.35(-0.00-0.80)	11.43(-0.00-26.45)	2.76(-0.00-6.16)	94.54(-0.01-214.30)	0.43(-0.00-0.95)	13.61(-0.00-30.66)	0.61(0.46-0.76)	0.45(0.29-0.60)
Kingdom of Cambodia	49.59(-0.01-126.43)	1787.95(-0.53-4533.21)	0.98(-0.00-2.45)	31.89(-0.01-81.26)	198.23(-0.04-459.85)	6766.38(-1.87-15723.07)	1.50(-0.00-3.46)	46.52(-0.01-107.77)	1.31(1.24-1.38)	1.15(1.07-1.23)
Kingdom of Denmark	237.06(-0.02-504.55)	6110.45(-1.09-13035.90)	3.07(-0.00-6.53)	86.18(-0.02-183.55)	181.23(-0.09-391.52)	3856.73(-1.52-8260.37)	1.49(-0.00-3.20)	36.80(-0.01-78.13)	-2.38(-2.48--2.28)	-2.76(-2.88--2.65)
Kingdom of Eswatini	3.93(-0.00-8.64)	130.59(-0.06-285.95)	1.37(-0.00-3.01)	37.31(-0.02-82.73)	12.29(-0.01-30.87)	412.63(-0.23-1056.90)	2.19(-0.00-5.47)	60.09(-0.03-151.41)	1.77(1.37-2.16)	1.78(1.32-2.25)
Kingdom of Lesotho	9.12(-0.00-21.28)	277.52(-0.11-653.57)	1.10(-0.00-2.56)	30.35(-0.01-71.15)	22.96(-0.01-56.58)	734.01(-0.45-1812.08)	2.18(-0.00-5.38)	60.17(-0.04-148.72)	2.97(2.54-3.40)	3.03(2.57-3.50)
Kingdom of Morocco	59.11(-0.02-129.31)	2139.66(-0.57-4791.79)	0.39(-0.00-0.84)	12.95(-0.00-28.71)	237.78(-0.10-567.73)	8504.79(-3.28-20366.21)	0.65(-0.00-1.53)	22.06(-0.01-52.58)	1.96(1.86-2.06)	1.96(1.85-2.06)
Kingdom of Norway	116.88(-0.05-249.23)	2921.80(-1.12-6217.83)	1.81(-0.00-3.86)	50.89(-0.02-108.91)	100.32(-0.04-213.41)	2342.82(-1.05-5001.75)	0.98(-0.00-2.09)	26.53(-0.01-56.30)	-2.18(-2.33--2.03)	-2.34(-2.53--2.15)
Kingdom of Saudi Arabia	27.65(-0.01-61.30)	1089.08(-0.37-2419.64)	0.38(-0.00-0.84)	12.30(-0.00-27.32)	162.32(-0.03-356.37)	7114.88(-1.14-15704.27)	0.53(-0.00-1.17)	18.43(-0.00-40.83)	1.06(0.77-1.36)	1.34(1.07-1.62)

Kingdom of Spain	922.66(-0.19-2003.60)	26134.84(-5.22-56543.17)	1.81(-0.00-3.92)	54.26(-0.01-117.30)	986.68(-0.59-2132.87)	23956.91(-15.55-51672.80)	1.00(-0.00-2.15)	29.10(-0.02-62.64)	-1.97(-2.03--1.90)	-2.08(-2.16--2.01)
Kingdom of Sweden	227.86(-0.04-486.59)	6000.67(-0.63-12928.73)	1.64(-0.00-3.50)	48.48(-0.01-104.56)	220.32(-0.09-479.58)	4849.91(-2.40-10701.81)	1.00(-0.00-2.18)	26.57(-0.01-58.55)	-1.32(-1.55--1.09)	-1.60(-1.87--1.34)
Kingdom of Thailand	310.24(-0.10-691.45)	11515.92(-4.20-25901.30)	0.76(-0.00-1.70)	25.55(-0.01-57.01)	1216.52(-0.20-2725.27)	38682.65(-8.61-86074.46)	1.15(-0.00-2.59)	38.14(-0.01-85.32)	1.41(1.18-1.65)	1.31(1.08-1.55)
Kingdom of the Netherlands	510.31(-0.17-1102.71)	13542.87(-4.15-29217.81)	2.62(-0.00-5.64)	72.73(-0.02-156.92)	516.72(-0.26-1114.44)	12232.15(-6.62-26237.79)	1.50(-0.00-3.22)	40.86(-0.02-87.55)	-2.04(-2.18--1.91)	-2.05(-2.19--1.92)
Kingdom of Tonga	1.32(-0.00-2.97)	44.25(-0.02-98.84)	2.36(-0.00-5.26)	72.28(-0.03-161.77)	2.21(-0.00-4.99)	67.18(-0.03-150.49)	2.72(-0.00-6.17)	79.61(-0.03-178.37)	0.41(0.35-0.47)	0.19(0.13-0.26)
Kyrgyz Republic	41.70(-0.01-89.79)	1369.35(-0.31-2959.33)	1.39(-0.00-2.99)	43.79(-0.01-94.28)	45.86(-0.03-101.94)	1573.34(-0.85-3496.49)	0.88(-0.00-1.95)	27.41(-0.01-60.70)	-1.55(-1.75--1.35)	-1.68(-1.85--1.51)
Lao People's Democratic Republic	20.80(-0.00-52.65)	747.92(-0.13-1918.65)	0.92(-0.00-2.28)	30.19(-0.00-76.52)	65.98(-0.04-150.27)	2352.83(-1.62-5391.78)	1.27(-0.00-2.86)	40.13(-0.02-91.29)	1.10(1.01-1.18)	0.98(0.89-1.07)
Lebanese Republic	30.26(-0.01-69.82)	963.91(-0.52-2251.81)	1.40(-0.00-3.19)	40.84(-0.02-94.60)	91.22(-0.05-194.79)	2682.45(-1.61-5800.60)	1.50(-0.00-3.22)	45.42(-0.03-98.60)	0.46(0.25-0.68)	0.63(0.40-0.87)

Malaysia	143.33(-0.05-315.05)	5146.06(-1.51-11457.62)	1.36(-0.00-2.98)	44.39(-0.01-98.95)	501.81(-0.19-1088.83)	17084.76(-6.47-37275.43)	1.69(-0.00-3.68)	54.19(-0.02-117.52)	0.63(0.52-0.74)	0.59(0.43-0.75)
Mongolia	4.43(-0.00-10.15)	154.69(-0.01-351.64)	0.40(-0.00-0.92)	12.86(-0.00-29.59)	11.52(-0.00-25.28)	411.09(-0.08-906.17)	0.45(-0.00-0.97)	13.80(-0.00-30.38)	0.09(-0.09-0.27)	-0.03(-0.22-0.16)
Montenegro	10.80(-0.00-24.40)	335.52(-0.12-755.63)	1.72(-0.00-3.90)	51.98(-0.02-117.00)	18.57(-0.01-41.11)	495.06(-0.25-1098.35)	2.02(-0.00-4.45)	54.18(-0.02-119.71)	0.61(0.47-0.76)	0.34(0.14-0.54)
New Zealand	92.01(-0.05-197.69)	2704.94(-2.06-5824.56)	2.44(-0.00-5.25)	73.07(-0.05-157.07)	104.25(-0.08-222.03)	2893.46(-2.20-5975.65)	1.30(-0.00-2.75)	39.70(-0.03-82.46)	-1.96(-2.03--1.89)	-1.88(-1.95--1.81)
North Macedonia	32.02(-0.02-70.07)	1067.74(-0.76-2338.99)	1.66(-0.00-3.64)	52.67(-0.04-115.48)	56.89(-0.02-126.50)	1577.31(-0.71-3495.46)	1.89(-0.00-4.22)	49.28(-0.02-109.84)	0.42(0.15-0.68)	-0.27(-0.47--0.07)
Northern Mariana Islands	0.34(-0.00-0.78)	13.47(-0.01-31.54)	1.67(-0.00-3.73)	45.85(-0.02-104.26)	0.99(-0.00-2.17)	31.89(-0.02-70.42)	1.98(-0.00-4.36)	54.88(-0.03-120.67)	0.56(0.38-0.74)	0.54(0.40-0.69)
Palestine	10.99(-0.00-26.54)	379.04(-0.03-925.73)	1.22(-0.00-2.89)	38.61(-0.00-94.50)	35.39(-0.00-78.38)	1264.95(-0.14-2777.19)	1.29(-0.00-2.86)	39.16(-0.00-85.59)	0.31(0.21-0.41)	0.12(0.05-0.20)
People's Democratic Republic of Algeria	58.77(-0.01-131.68)	2087.04(-0.25-4614.27)	0.49(-0.00-1.09)	15.00(-0.00-33.27)	204.22(-0.04-463.65)	7173.22(-1.61-16329.79)	0.55(-0.00-1.26)	17.16(-0.00-39.01)	0.71(0.56-0.85)	0.54(0.44-0.65)
People's Republic of Bangladesh	79.30(-0.01-184.81)	3331.46(-0.36-7927.69)	0.13(-0.00-0.31)	5.29(-0.00-12.34)	359.09(-0.03-826.34)	14691.77(-1.19-33525.00)	0.23(-0.00-0.54)	9.25(-0.00-21.10)	1.57(1.41-1.73)	1.64(1.47-1.80)

People's Republic of China	5575.79(-1.62-12297.65)	201432.38(-59.68-442861.06)	0.64(-0.00-1.40)	20.49(-0.01-45.17)	12530.06(-6.57-27433.52)	414541.14(-239.85-918598.73)	0.60(-0.00-1.32)	19.95(-0.01-44.22)	-0.47(-0.60--0.34)	-0.33(-0.44--0.22)
Plurinational State of Bolivia	38.70(-0.01-95.91)	1308.27(-0.31-3234.24)	1.14(-0.00-2.79)	34.68(-0.01-86.12)	122.57(-0.05-277.40)	3905.58(-1.55-8800.38)	1.31(-0.00-2.98)	38.56(-0.01-86.95)	0.32(0.27-0.37)	0.19(0.13-0.24)
Portuguese Republic	249.64(-0.01-529.48)	7163.94(-0.12-15179.03)	1.96(-0.00-4.18)	57.46(-0.00-121.67)	287.84(-0.19-626.54)	6816.79(-4.62-14786.48)	1.20(-0.00-2.61)	34.95(-0.03-75.77)	-1.72(-1.80--1.65)	-1.70(-1.80--1.60)
Principality of Andorra	0.92(-0.00-2.08)	28.88(-0.02-66.56)	1.65(-0.00-3.71)	48.71(-0.03-111.70)	1.91(-0.00-4.21)	53.07(-0.04-115.61)	1.21(-0.00-2.66)	35.89(-0.03-78.31)	-0.58(-0.76--0.39)	-0.58(-0.76--0.40)
Principality of Monaco	1.67(-0.00-3.90)	40.37(-0.02-93.51)	2.55(-0.00-5.85)	73.22(-0.04-169.01)	2.40(-0.00-5.39)	56.60(-0.04-126.15)	2.59(-0.00-5.82)	76.46(-0.05-171.32)	0.13(0.04-0.22)	0.22(0.12-0.32)
Puerto Rico	48.72(-0.02-101.28)	1517.81(-0.62-3165.40)	1.39(-0.00-2.90)	42.79(-0.02-89.43)	79.44(-0.02-172.22)	1995.55(-0.62-4318.98)	1.19(-0.00-2.57)	35.73(-0.01-76.91)	-0.60(-0.72--0.47)	-0.70(-0.83--0.57)
Republic of Albania	14.35(-0.01-31.10)	465.10(-0.34-1014.36)	0.69(-0.00-1.48)	19.73(-0.01-43.01)	31.30(-0.02-68.98)	847.25(-0.49-1863.61)	0.78(-0.00-1.71)	22.18(-0.01-48.75)	0.78(0.60-0.97)	0.80(0.59-1.02)
Republic of Angola	39.05(-0.01-88.22)	1437.38(-0.39-3233.10)	0.87(-0.00-2.00)	27.20(-0.01-61.42)	187.65(-0.08-423.72)	6856.71(-2.62-15576.68)	1.40(-0.00-3.25)	42.09(-0.02-95.24)	1.67(1.57-1.76)	1.57(1.47-1.68)
Republic of Armenia	61.88(-0.03-130.78)	2103.67(-0.97-4460.05)	2.18(-0.00-4.60)	70.10(-0.04-148.48)	68.59(-0.03-145.15)	1818.99(-0.84-3823.32)	1.59(-0.00-3.36)	42.95(-0.02-90.31)	-1.04(-1.35--0.73)	-1.62(-1.96--1.28)

Republic of Austria	256.03(-0.10-549.83)	6528.45(-3.13-14076.26)	2.24(-0.00-4.84)	61.75(-0.04-132.59)	237.26(-0.13-510.71)	5240.04(-3.17-11211.31)	1.26(-0.00-2.68)	32.71(-0.02-69.54)	-1.80(-1.86--1.74)	-1.95(-2.03--1.87)
Republic of Azerbaijan	67.98(-0.03-146.82)	2351.22(-0.94-5113.28)	1.32(-0.00-2.86)	42.45(-0.02-91.84)	118.31(-0.07-267.66)	4036.05(-2.27-9180.51)	1.06(-0.00-2.38)	33.41(-0.02-75.91)	-0.49(-0.67--0.31)	-0.69(-0.83--0.56)
Republic of Belarus	170.81(-0.01-372.46)	5436.17(-0.11-11907.27)	1.35(-0.00-2.93)	43.74(-0.00-95.41)	186.87(-0.10-415.90)	5288.38(-2.66-11637.99)	1.20(-0.00-2.66)	35.58(-0.02-77.84)	-1.31(-1.61--1.01)	-1.56(-1.85--1.27)
Republic of Benin	14.06(-0.00-30.61)	475.42(-0.04-1032.77)	0.67(-0.00-1.46)	20.79(-0.00-45.33)	41.33(-0.00-95.95)	1430.21(-0.10-3356.54)	0.74(-0.00-1.69)	21.51(-0.00-49.91)	0.25(0.09-0.41)	0.01(-0.17-0.19)
Republic of Botswana	7.33(-0.00-16.94)	247.98(-0.09-576.90)	1.33(-0.00-3.07)	37.73(-0.02-87.82)	24.93(-0.01-57.11)	817.86(-0.49-1928.77)	1.69(-0.00-3.75)	45.24(-0.02-103.98)	1.25(0.91-1.59)	1.06(0.70-1.42)
Republic of Bulgaria	178.94(-0.10-388.77)	5679.09(-4.03-12306.56)	1.56(-0.00-3.37)	49.17(-0.04-106.21)	241.05(-0.18-532.85)	6199.11(-5.02-13828.34)	1.81(-0.00-4.01)	51.52(-0.04-114.79)	0.69(0.56-0.82)	0.31(0.19-0.43)
Republic of Burundi	15.04(-0.00-33.79)	525.34(-0.03-1174.63)	0.61(-0.00-1.37)	18.97(-0.00-42.23)	22.54(-0.00-53.65)	824.02(-0.05-1937.11)	0.42(-0.00-1.00)	12.26(-0.00-29.40)	-1.54(-1.93--1.14)	-1.74(-2.17--1.31)
Republic of Cabo Verde	2.29(-0.00-5.11)	69.95(-0.02-157.20)	1.07(-0.00-2.41)	34.30(-0.01-77.25)	4.98(-0.00-11.04)	143.68(-0.06-326.81)	1.09(-0.00-2.42)	28.99(-0.01-65.64)	0.29(0.01-0.56)	-0.32(-0.60--0.03)
Republic of Cameroon	49.65(-0.02-106.71)	1702.85(-0.61-3634.25)	1.05(-0.00-2.25)	31.40(-0.01-67.29)	170.87(-0.04-398.42)	6048.50(-1.47-14091.96)	1.24(-0.00-2.82)	36.19(-0.01-84.37)	0.49(0.41-0.56)	0.38(0.30-0.47)

Republic of Chad	19.13(-0.01-42.44)	630.65(-0.34-1403.21)	0.67(-0.00-1.47)	20.31(-0.01-44.71)	52.12(-0.02-116.45)	1855.87(-0.79-4225.16)	0.81(-0.00-1.79)	24.47(-0.01-54.78)	0.67(0.64-0.70)	0.63(0.60-0.67)
Republic of Chile	133.18(-0.07-279.85)	4018.53(-2.32-8507.56)	1.34(-0.00-2.81)	37.52(-0.02-79.35)	243.10(-0.14-526.77)	6507.78(-3.27-13979.27)	0.96(-0.00-2.07)	26.59(-0.01-57.09)	-0.88(-0.97--0.79)	-0.91(-1.01--0.81)
Republic of Colombia	193.30(-0.06-414.40)	6562.48(-1.54-14141.58)	1.03(-0.00-2.21)	31.41(-0.01-67.75)	580.29(-0.30-1279.19)	18418.65(-9.52-40844.60)	1.05(-0.00-2.32)	33.49(-0.02-74.26)	-0.01(-0.24-0.22)	0.16(-0.07-0.40)
Republic of Costa Rica	16.12(-0.01-34.56)	532.97(-0.21-1128.33)	0.89(-0.00-1.90)	27.21(-0.01-58.17)	67.03(-0.04-143.44)	2068.53(-1.31-4402.84)	1.22(-0.00-2.60)	37.62(-0.02-80.15)	1.11(0.96-1.26)	1.09(0.92-1.25)
Republic of Côte d'Ivoire	49.30(-0.03-107.59)	1741.31(-1.01-3831.21)	1.13(-0.00-2.41)	32.65(-0.02-70.98)	181.41(-0.04-402.00)	6366.37(-2.15-14328.71)	1.47(-0.00-3.24)	42.35(-0.01-93.59)	0.96(0.86-1.06)	0.96(0.86-1.06)
Republic of Croatia	113.17(-0.05-245.91)	3149.14(-1.29-6889.21)	1.95(-0.00-4.23)	51.97(-0.03-113.54)	130.46(-0.05-275.50)	2975.72(-1.19-6379.32)	1.47(-0.00-3.11)	38.03(-0.01-82.42)	-0.80(-0.99--0.60)	-1.00(-1.18--0.82)
Republic of Cuba	135.58(-0.10-285.81)	4136.74(-2.73-8740.46)	1.34(-0.00-2.83)	40.23(-0.03-84.96)	250.98(-0.13-554.73)	6509.34(-3.29-14436.04)	1.29(-0.00-2.84)	35.68(-0.02-78.81)	-0.04(-0.16-0.08)	-0.35(-0.45--0.25)
Republic of Cyprus	13.83(-0.00-29.91)	398.93(-0.06-859.09)	1.89(-0.00-4.06)	50.54(-0.01-109.06)	29.13(-0.02-63.03)	777.83(-0.42-1697.51)	1.56(-0.00-3.37)	40.78(-0.02-88.39)	-0.42(-0.56--0.29)	-0.55(-0.70--0.40)
Republic of Djibouti	2.02(-0.00-4.44)	73.86(-0.03-164.00)	1.33(-0.00-2.97)	38.30(-0.02-84.17)	11.12(-0.00-26.37)	397.53(-0.19-936.75)	1.64(-0.00-3.86)	45.23(-0.02-106.59)	0.66(0.62-0.70)	0.50(0.44-0.55)

Republic of Ecuador	36.08(-0.02-76.56)	1215.17(-0.76-2562.92)	0.64(-0.00-1.36)	19.53(-0.01-41.47)	150.57(-0.08-323.60)	4615.73(-2.58-9941.18)	0.92(-0.00-1.98)	27.19(-0.02-58.52)	1.29(1.08-1.50)	1.21(0.99-1.44)
Republic of El Salvador	13.90(-0.00-30.89)	486.97(-0.04-1088.97)	0.44(-0.00-0.98)	14.73(-0.00-32.84)	50.59(-0.01-117.32)	1662.86(-0.26-3798.77)	0.82(-0.00-1.91)	27.43(-0.00-62.65)	1.99(1.79-2.20)	2.02(1.82-2.22)
Republic of Equatorial Guinea	2.20(-0.00-5.24)	77.07(-0.02-186.13)	1.06(-0.00-2.45)	32.78(-0.01-78.06)	11.06(-0.00-27.88)	399.52(-0.14-1001.86)	1.92(-0.00-4.82)	56.18(-0.02-141.60)	2.11(1.99-2.24)	1.89(1.77-2.02)
Republic of Estonia	34.71(-0.01-73.41)	1009.20(-0.09-2154.60)	1.73(-0.00-3.67)	51.18(-0.00-109.45)	33.99(-0.02-76.13)	768.68(-0.50-1715.71)	1.25(-0.00-2.80)	32.83(-0.02-73.33)	-1.29(-1.45--1.14)	-1.73(-1.92--1.54)
Republic of Fiji	9.05(-0.00-20.27)	337.87(-0.10-766.26)	2.23(-0.00-5.02)	68.90(-0.02-154.82)	21.36(-0.01-46.64)	695.08(-0.43-1524.76)	2.88(-0.00-6.30)	81.05(-0.05-177.49)	0.88(0.78-0.98)	0.65(0.50-0.79)
Republic of Finland	124.12(-0.06-262.44)	3461.00(-1.19-7365.03)	1.80(-0.00-3.82)	52.22(-0.01-111.27)	142.15(-0.06-305.75)	3280.51(-1.45-7136.64)	1.13(-0.00-2.45)	31.95(-0.01-69.31)	-1.47(-1.53--1.42)	-1.57(-1.65--1.49)
Republic of Ghana	67.98(-0.00-151.51)	2514.49(-0.23-5601.25)	0.96(-0.00-2.12)	30.60(-0.00-68.33)	236.25(-0.03-550.59)	8410.61(-1.05-19346.71)	1.28(-0.00-2.96)	38.48(-0.00-89.05)	0.96(0.88-1.03)	0.73(0.65-0.81)
Republic of Guatemala	12.03(-0.00-25.94)	439.44(-0.03-944.87)	0.33(-0.00-0.71)	10.06(-0.00-21.68)	64.63(-0.01-139.65)	2238.31(-0.44-4857.89)	0.56(-0.00-1.22)	18.18(-0.00-39.57)	1.73(1.42-2.04)	1.90(1.60-2.21)
Republic of Guinea	19.46(-0.00-44.81)	655.37(-0.05-1480.66)	0.56(-0.00-1.29)	17.87(-0.00-40.66)	61.48(-0.01-142.69)	2165.22(-0.47-4975.30)	0.98(-0.00-2.26)	30.84(-0.01-71.54)	1.92(1.79-2.05)	1.87(1.76-1.98)

Republic of Guinea-Bissau	4.69(-0.00-10.56)	172.20(-0.05-388.78)	1.07(-0.00-2.42)	34.30(-0.01-77.33)	12.56(-0.01-29.30)	475.65(-0.23-1106.28)	1.48(-0.00-3.45)	45.28(-0.02-106.48)	1.10(1.08-1.12)	0.93(0.91-0.95)
Republic of Guyana	4.16(-0.00-9.08)	145.15(-0.01-314.96)	1.01(-0.00-2.20)	31.47(-0.00-68.46)	8.71(-0.00-20.01)	298.77(-0.02-693.90)	1.28(-0.00-2.95)	41.20(-0.00-95.24)	0.99(0.75-1.24)	1.13(0.86-1.40)
Republic of Haiti	46.79(-0.00-126.89)	1676.15(-0.14-4616.92)	1.35(-0.00-3.55)	42.54(-0.00-115.35)	130.13(-0.02-322.02)	4695.33(-0.70-11872.53)	1.59(-0.00-3.87)	49.62(-0.01-123.64)	0.65(0.49-0.81)	0.62(0.46-0.78)
Republic of Honduras	11.55(-0.00-27.00)	416.51(-0.04-950.04)	0.52(-0.00-1.22)	16.83(-0.00-39.07)	61.07(-0.02-145.36)	2038.22(-0.79-4723.70)	0.91(-0.00-2.17)	27.92(-0.01-65.36)	1.85(1.66-2.03)	1.64(1.48-1.79)
Republic of Iceland	5.50(-0.00-11.64)	153.42(-0.11-327.15)	1.97(-0.00-4.17)	57.73(-0.04-122.83)	7.11(-0.00-15.03)	178.81(-0.11-382.74)	1.23(-0.00-2.58)	34.65(-0.02-74.44)	-1.51(-1.63--1.39)	-1.73(-1.83--1.62)
Republic of India	1047.37(-0.02-2367.42)	37680.23(-0.58-85834.15)	0.20(-0.00-0.46)	6.47(-0.00-14.67)	4053.12(-0.07-9290.75)	133041.43(-2.55-306554.63)	0.33(-0.00-0.76)	10.07(-0.00-23.21)	1.59(1.34-1.83)	1.43(1.17-1.68)
Republic of Indonesia	683.70(-0.02-1719.38)	25621.15(-0.86-63897.67)	0.60(-0.00-1.51)	20.06(-0.00-50.33)	2423.52(-0.10-6275.15)	86420.32(-3.78-222447.10)	0.90(-0.00-2.29)	29.04(-0.00-74.70)	1.20(1.08-1.31)	1.05(0.93-1.17)
Republic of Iraq	63.63(-0.01-142.28)	2376.96(-0.28-5323.66)	0.71(-0.00-1.60)	24.71(-0.00-55.49)	229.03(-0.02-554.74)	8804.50(-0.75-21272.52)	0.81(-0.00-1.96)	27.46(-0.00-66.61)	0.62(0.24-1.00)	0.54(0.19-0.89)
Republic of Italy	1783.38(-0.91-3778.17)	49631.26(-26.50-104875.13)	2.13(-0.00-4.51)	62.77(-0.03-132.81)	2000.45(-0.73-4297.59)	44991.09(-19.31-95492.76)	1.35(-0.00-2.87)	37.58(-0.02-79.99)	-1.54(-1.60--1.48)	-1.73(-1.80--1.66)

Republic of Kazakhstan	223.89(-0.06-486.20)	7326.25(-1.87-15826.73)	1.72(-0.00-3.75)	53.09(-0.02-114.91)	188.46(-0.13-423.01)	6039.48(-4.63-13534.51)	1.03(-0.00-2.32)	30.71(-0.02-68.84)	-1.05(-1.44--0.66)	-1.32(-1.67--0.97)
Republic of Kenya	73.60(-0.02-171.27)	2521.13(-0.59-5866.23)	0.83(-0.00-1.95)	25.10(-0.01-58.57)	353.27(-0.07-821.35)	12328.05(-2.77-28993.72)	1.44(-0.00-3.26)	41.70(-0.01-97.41)	2.08(1.96-2.19)	1.89(1.80-1.98)
Republic of Kiribati	0.66(-0.00-1.49)	23.61(-0.01-53.97)	1.70(-0.00-3.77)	51.64(-0.02-117.23)	1.77(-0.00-3.94)	62.19(-0.02-139.16)	2.38(-0.00-5.23)	69.64(-0.02-155.20)	1.19(1.09-1.28)	1.09(1.00-1.17)
Republic of Korea	138.19(-0.08-297.03)	5482.39(-3.14-11686.06)	0.40(-0.00-0.85)	14.03(-0.01-29.84)	389.39(-0.22-810.57)	13320.28(-8.11-28269.65)	0.45(-0.00-0.92)	16.04(-0.01-34.19)	0.29(0.19-0.40)	0.52(0.42-0.61)
Republic of Latvia	57.19(-0.02-124.34)	1722.88(-0.57-3744.61)	1.64(-0.00-3.56)	50.50(-0.01-109.81)	57.79(-0.04-125.69)	1366.87(-0.84-2949.49)	1.48(-0.00-3.24)	40.33(-0.02-87.61)	-0.34(-0.53--0.14)	-0.84(-1.07--0.62)
Republic of Liberia	6.94(-0.00-15.05)	231.50(-0.01-502.90)	0.59(-0.00-1.27)	17.86(-0.00-39.03)	18.89(-0.00-44.02)	700.69(-0.05-1645.08)	0.74(-0.00-1.74)	22.57(-0.00-52.52)	1.06(0.76-1.37)	1.00(0.70-1.30)
Republic of Lithuania	64.54(-0.02-139.29)	1982.36(-0.49-4258.91)	1.46(-0.00-3.14)	45.63(-0.01-97.86)	77.70(-0.04-174.21)	1890.05(-1.05-4258.15)	1.39(-0.00-3.15)	39.11(-0.02-88.53)	-0.16(-0.37-0.06)	-0.55(-0.78--0.32)
Republic of Madagascar	64.22(-0.03-139.75)	2249.09(-0.94-4967.89)	1.18(-0.00-2.59)	36.58(-0.02-79.78)	163.56(-0.06-380.53)	6177.38(-2.11-14358.88)	1.26(-0.00-2.92)	38.28(-0.01-89.23)	0.12(-0.10-0.34)	0.04(-0.19-0.26)
Republic of Malawi	21.35(-0.00-47.03)	755.62(-0.05-1686.42)	0.51(-0.00-1.12)	15.76(-0.00-34.89)	93.55(-0.01-214.48)	3332.88(-0.33-7744.40)	1.15(-0.00-2.63)	33.94(-0.00-77.63)	3.06(2.96-3.16)	2.87(2.77-2.97)

Republic of Maldives	0.16(-0.00-0.49)	5.77(-0.00-18.29)	0.15(-0.00-0.44)	4.96(-0.00-15.39)	1.21(-0.00-2.70)	43.20(-0.00-96.57)	0.31(-0.00-0.68)	9.20(-0.00-20.74)	2.14(1.49-2.79)	1.85(1.15-2.56)
Republic of Mali	43.29(-0.03-91.12)	1497.83(-1.18-3147.62)	1.00(-0.00-2.11)	31.21(-0.02-65.39)	112.41(-0.05-254.09)	3922.06(-1.38-8924.34)	1.15(-0.00-2.58)	35.13(-0.01-79.23)	0.38(0.29-0.46)	0.29(0.20-0.38)
Republic of Malta	11.08(-0.00-23.81)	312.57(-0.06-669.64)	2.64(-0.00-5.69)	73.40(-0.01-157.52)	13.62(-0.01-30.33)	327.83(-0.19-722.11)	1.47(-0.00-3.25)	41.72(-0.03-91.31)	-2.21(-2.36--2.05)	-2.07(-2.18--1.96)
Republic of Mauritius	6.43(-0.00-13.45)	215.60(-0.05-450.91)	0.85(-0.00-1.79)	25.75(-0.00-53.79)	29.61(-0.01-63.36)	904.63(-0.48-1920.30)	1.64(-0.00-3.50)	50.61(-0.03-107.07)	1.56(1.23-1.90)	1.58(1.24-1.92)
Republic of Moldova	74.39(-0.04-160.99)	2446.38(-1.71-5263.29)	1.68(-0.00-3.62)	53.36(-0.04-114.83)	79.83(-0.01-180.63)	2305.44(-0.47-5202.50)	1.36(-0.00-3.08)	40.53(-0.01-91.36)	-0.24(-0.50-0.01)	-0.61(-0.83--0.38)
Republic of Mozambique	38.97(-0.00-87.52)	1362.85(-0.08-3049.76)	0.64(-0.00-1.46)	18.41(-0.00-41.15)	143.78(-0.01-342.52)	5106.94(-0.36-11980.81)	1.24(-0.00-2.98)	34.72(-0.00-82.51)	2.42(2.31-2.53)	2.25(2.15-2.36)
Republic of Namibia	9.21(-0.01-20.88)	309.28(-0.28-701.02)	1.40(-0.00-3.12)	41.26(-0.04-94.19)	36.40(-0.02-86.38)	1216.18(-0.73-2914.37)	2.49(-0.00-5.89)	73.01(-0.04-173.39)	2.17(2.04-2.30)	2.14(2.00-2.28)
Republic of Nauru	0.10(-0.00-0.26)	3.91(-0.00-10.15)	1.89(-0.00-4.82)	61.34(-0.03-157.87)	0.18(-0.00-0.46)	6.44(-0.00-16.84)	2.84(-0.00-7.28)	86.55(-0.04-223.99)	1.37(1.31-1.43)	1.17(1.09-1.24)
Republic of Nicaragua	6.97(-0.00-15.37)	258.77(-0.02-567.18)	0.40(-0.00-0.89)	13.27(-0.00-29.23)	28.11(-0.00-62.62)	981.54(-0.10-2184.96)	0.54(-0.00-1.20)	17.51(-0.00-38.95)	1.31(1.15-1.48)	1.20(1.05-1.35)
Republic of Niue	0.05(-0.00-0.10)	1.24(-0.00-2.78)	2.12(-0.00-4.66)	58.86(-0.01-131.49)	0.05(-0.00-0.12)	1.46(-0.00-3.33)	2.54(-0.00-5.67)	71.67(-0.02-161.91)	0.42(0.35-0.50)	0.37(0.29-0.46)

Republic of Palau	0.29(-0.00-0.66)	9.15(-0.00-21.13)	3.02(-0.00-6.84)	82.19(-0.03-190.53)	0.59(-0.00-1.30)	17.34(-0.01-38.18)	2.94(-0.00-6.51)	74.47(-0.03-164.41)	0.04(-0.07-0.15)	-0.22(-0.33--0.10)
Republic of Panama	12.42(-0.00-26.33)	405.25(-0.09-859.66)	0.81(-0.00-1.71)	24.45(-0.01-51.90)	45.47(-0.02-97.22)	1461.48(-0.82-3145.74)	1.02(-0.00-2.18)	33.07(-0.02-71.27)	0.92(0.80-1.04)	1.14(1.03-1.26)
Republic of Paraguay	20.93(-0.02-45.99)	683.87(-0.49-1503.61)	0.90(-0.00-2.00)	27.24(-0.02-59.57)	77.65(-0.04-178.06)	2406.46(-1.49-5567.24)	1.31(-0.00-3.01)	38.09(-0.02-88.02)	1.22(1.05-1.40)	1.06(0.87-1.25)
Republic of Peru	104.78(-0.01-230.96)	3601.97(-0.43-8028.80)	0.81(-0.00-1.79)	25.79(-0.00-57.37)	276.10(-0.06-646.19)	8815.42(-2.01-20217.81)	0.80(-0.00-1.87)	24.83(-0.01-56.94)	-0.46(-0.68--0.25)	-0.53(-0.74--0.31)
Republic of Poland	691.62(-0.34-1459.99)	20233.05(-9.88-42975.48)	1.64(-0.00-3.45)	47.32(-0.02-100.60)	1174.86(-0.49-2458.36)	27204.27(-12.69-56629.34)	1.65(-0.00-3.44)	41.91(-0.02-87.34)	-0.23(-0.37--0.10)	-0.57(-0.69--0.45)
Republic of Rwanda	33.75(-0.00-81.92)	1230.81(-0.07-3009.26)	1.09(-0.00-2.59)	34.49(-0.00-84.01)	95.04(-0.01-218.85)	3306.61(-0.39-7693.82)	1.42(-0.00-3.23)	40.97(-0.00-94.31)	0.80(0.60-1.00)	0.42(0.21-0.63)
Republic of San Marino	0.52(-0.00-1.11)	13.15(-0.01-28.34)	1.52(-0.00-3.22)	41.16(-0.03-89.29)	0.60(-0.00-1.41)	14.60(-0.01-34.64)	0.80(-0.00-1.91)	24.12(-0.01-56.83)	-1.38(-1.65--1.10)	-0.96(-1.24--0.69)
Republic of Senegal	25.93(-0.00-55.06)	888.78(-0.18-1932.24)	0.76(-0.00-1.60)	23.29(-0.00-50.13)	92.11(-0.03-205.38)	3099.99(-0.83-6950.37)	1.12(-0.00-2.53)	33.20(-0.01-73.97)	1.35(1.22-1.49)	1.22(1.07-1.37)
Republic of Serbia	225.32(-0.11-489.98)	6784.23(-3.44-14729.50)	2.16(-0.00-4.72)	59.86(-0.03-129.81)	318.01(-0.14-700.57)	7994.58(-3.68-17595.12)	1.99(-0.00-4.37)	54.23(-0.03-119.27)	-0.58(-0.72--0.43)	-0.60(-0.77--0.43)
Republic of Seychelles	0.52(-0.00-1.15)	16.63(-0.00-37.36)	0.93(-0.00-2.06)	29.71(-0.00-66.67)	1.58(-0.00-3.36)	50.82(-0.01-108.52)	1.32(-0.00-2.80)	39.78(-0.01-84.52)	1.10(0.74-1.46)	0.95(0.63-1.28)

Republic of Sierra Leone	6.94(-0.00-15.86)	234.28(-0.02-551.90)	0.33(-0.00-0.75)	10.04(-0.00-23.29)	26.65(-0.00-61.22)	960.95(-0.07-2202.68)	0.62(-0.00-1.42)	19.34(-0.00-44.26)	2.54(2.36-2.72)	2.59(2.40-2.79)
Republic of Singapore	26.80(-0.02-57.12)	977.23(-0.73-2079.25)	1.09(-0.00-2.30)	35.21(-0.03-74.86)	66.19(-0.05-142.12)	2069.45(-1.56-4460.54)	0.76(-0.00-1.64)	23.77(-0.02-51.36)	-0.91(-1.07--0.74)	-1.04(-1.22--0.86)
Republic of Slovenia	43.76(-0.01-94.10)	1208.11(-0.14-2625.96)	1.79(-0.00-3.86)	49.99(-0.01-108.71)	59.20(-0.04-128.75)	1230.95(-0.69-2648.64)	1.26(-0.00-2.73)	30.45(-0.02-65.69)	-1.42(-1.66--1.19)	-1.84(-2.07--1.60)
Republic of South Africa	293.12(-0.11-642.49)	9804.00(-3.88-21862.11)	1.37(-0.00-3.00)	40.24(-0.02-88.65)	891.71(-0.30-1940.82)	27596.41(-10.48-60691.74)	1.95(-0.00-4.23)	53.51(-0.02-117.19)	1.49(1.28-1.70)	1.42(1.18-1.67)
Republic of South Sudan	22.80(-0.00-54.11)	753.12(-0.11-1793.28)	0.88(-0.00-2.07)	25.75(-0.00-61.16)	46.61(-0.01-108.69)	1661.21(-0.39-3897.04)	1.08(-0.00-2.53)	31.76(-0.01-73.84)	0.63(0.40-0.85)	0.62(0.36-0.87)
Republic of Sudan	35.03(-0.01-88.53)	1307.71(-0.43-3422.45)	0.34(-0.00-0.83)	11.56(-0.00-29.90)	121.21(-0.01-307.43)	4817.01(-0.59-12488.02)	0.50(-0.00-1.23)	17.27(-0.00-43.78)	1.35(1.17-1.53)	1.44(1.26-1.62)
Republic of Suriname	2.78(-0.00-6.08)	91.26(-0.01-200.77)	1.04(-0.00-2.28)	31.99(-0.00-70.18)	7.26(-0.00-16.41)	227.60(-0.03-509.82)	1.14(-0.00-2.57)	34.83(-0.00-77.57)	0.63(0.48-0.78)	0.54(0.38-0.70)
Republic of Tajikistan	29.04(-0.01-64.41)	1011.95(-0.40-2238.80)	1.01(-0.00-2.27)	33.23(-0.01-73.89)	49.43(-0.02-119.71)	1845.58(-0.70-4581.40)	0.71(-0.00-1.66)	23.66(-0.01-57.52)	-1.20(-1.40--1.01)	-1.16(-1.36--0.95)
Republic of the Congo	18.14(-0.00-47.79)	627.92(-0.14-1684.52)	1.62(-0.00-4.17)	49.67(-0.01-131.80)	62.73(-0.02-160.74)	2296.88(-1.01-5898.64)	2.00(-0.00-5.07)	61.01(-0.02-156.51)	0.64(0.53-0.75)	0.59(0.47-0.72)

Republic of the Gambia	1.32(-0.00-2.91)	45.73(-0.00-99.85)	0.35(-0.00-0.77)	10.41(-0.00-22.58)	5.61(-0.00-13.13)	194.91(-0.02-455.35)	0.52(-0.00-1.22)	15.75(-0.00-36.79)	1.14(0.90-1.38)	1.15(0.88-1.43)
Republic of the Marshall Islands	0.26(-0.00-0.62)	9.23(-0.00-22.29)	1.51(-0.00-3.52)	44.52(-0.01-106.46)	0.80(-0.00-1.99)	30.04(-0.01-75.16)	2.06(-0.00-5.05)	64.30(-0.02-160.03)	0.89(0.79-1.00)	1.10(0.98-1.23)
Republic of the Niger	17.75(-0.01-38.52)	634.42(-0.39-1397.30)	0.59(-0.00-1.27)	17.53(-0.01-38.02)	59.42(-0.04-138.39)	2029.01(-1.17-4813.56)	0.68(-0.00-1.59)	19.87(-0.01-46.53)	0.45(0.37-0.54)	0.32(0.22-0.42)
Republic of the Philippines	415.61(-0.11-912.74)	14856.89(-4.29-32804.31)	1.30(-0.00-2.80)	39.60(-0.01-87.25)	1531.51(-0.29-3413.53)	53020.94(-10.06-118319.33)	1.73(-0.00-3.83)	54.33(-0.01-120.98)	0.90(0.80-0.99)	1.02(0.95-1.09)
Republic of the Union of Myanmar	198.39(-0.02-469.23)	7778.22(-0.68-18434.98)	0.74(-0.00-1.76)	26.86(-0.00-63.55)	701.19(-0.25-1569.26)	24695.19(-10.23-56059.68)	1.34(-0.00-2.97)	44.38(-0.02-100.64)	2.14(1.86-2.43)	1.78(1.48-2.09)
Republic of Trinidad and Tobago	15.28(-0.00-32.52)	486.84(-0.14-1046.88)	1.87(-0.00-4.01)	54.63(-0.02-117.61)	33.90(-0.01-77.31)	1026.05(-0.21-2369.33)	1.80(-0.00-4.10)	55.14(-0.01-127.53)	0.08(-0.12-0.29)	0.17(-0.02-0.36)
Republic of Tunisia	32.63(-0.01-70.50)	1121.21(-0.37-2443.72)	0.64(-0.00-1.40)	20.07(-0.01-43.66)	103.24(-0.03-229.39)	3473.54(-1.38-7706.11)	0.77(-0.00-1.71)	24.83(-0.01-55.05)	0.46(0.36-0.56)	0.58(0.48-0.68)
Republic of Turkey	150.05(-0.05-331.47)	5290.79(-1.69-11670.69)	0.40(-0.00-0.88)	12.94(-0.00-28.58)	843.44(-0.27-1864.96)	27662.61(-9.91-61245.67)	0.90(-0.00-1.99)	28.45(-0.01-62.99)	3.92(3.16-4.70)	3.78(3.02-4.54)
Republic of Uganda	80.26(-0.01-170.00)	2653.65(-0.18-5770.84)	1.21(-0.00-2.61)	35.09(-0.00-74.77)	317.15(-0.10-720.59)	11100.57(-3.87-25172.32)	1.95(-0.00-4.41)	57.50(-0.02-130.08)	1.08(0.80-1.35)	1.05(0.74-1.35)

Republic of Uzbekistan	122.08(-0.08-267.57)	4172.46(-2.55-9134.27)	1.03(-0.00-2.25)	33.19(-0.02-72.75)	252.94(-0.11-555.50)	9185.54(-4.08-20243.78)	0.84(-0.00-1.85)	28.03(-0.01-61.50)	-0.53(-0.75--0.31)	-0.48(-0.68--0.27)
Republic of Vanuatu	0.62(-0.00-1.43)	22.66(-0.01-52.33)	0.94(-0.00-2.12)	28.00(-0.02-64.37)	2.72(-0.00-6.30)	97.02(-0.05-227.08)	1.47(-0.00-3.34)	44.32(-0.02-103.45)	1.32(1.22-1.42)	1.28(1.15-1.40)
Republic of Yemen	15.70(-0.00-36.47)	580.61(-0.05-1359.79)	0.29(-0.00-0.66)	9.28(-0.00-21.69)	80.87(-0.01-186.34)	3109.15(-0.35-7273.25)	0.47(-0.00-1.07)	15.71(-0.00-36.12)	1.98(1.78-2.18)	2.08(1.88-2.28)
Republic of Zambia	36.22(-0.01-84.25)	1329.02(-0.28-3068.24)	1.16(-0.00-2.65)	35.80(-0.01-83.46)	174.64(-0.05-432.96)	6652.35(-2.37-17005.89)	2.11(-0.00-5.08)	64.58(-0.02-159.24)	2.12(1.79-2.45)	2.06(1.71-2.42)
Republic of Zimbabwe	45.63(-0.01-102.56)	1493.29(-0.33-3398.98)	1.12(-0.00-2.47)	31.49(-0.01-71.06)	165.37(-0.05-374.83)	5857.92(-1.93-13502.15)	2.16(-0.00-4.80)	64.99(-0.02-148.60)	2.96(2.25-3.67)	3.33(2.51-4.14)
Romania	363.37(-0.29-777.49)	11596.00(-9.87-24944.52)	1.34(-0.00-2.86)	42.34(-0.03-90.98)	566.21(-0.25-1217.70)	14265.31(-6.14-30999.63)	1.57(-0.00-3.38)	43.87(-0.02-95.32)	0.39(0.25-0.52)	-0.02(-0.15-0.10)
Russian Federation	2370.59(-1.10-5029.03)	75300.06(-36.27-160710.16)	1.34(-0.00-2.84)	42.37(-0.02-90.43)	3445.02(-1.57-7251.34)	94209.61(-40.69-197619.21)	1.47(-0.00-3.11)	42.12(-0.02-88.35)	-0.10(-0.40-0.20)	-0.45(-0.73--0.17)
Saint Kitts and Nevis	1.10(-0.00-2.38)	30.50(-0.01-65.99)	3.17(-0.00-6.86)	92.16(-0.05-198.04)	1.46(-0.00-3.18)	42.40(-0.03-91.72)	2.22(-0.00-4.83)	57.02(-0.03-123.43)	-0.60(-0.81--0.39)	-1.14(-1.39--0.90)
Saint Lucia	1.92(-0.00-4.12)	58.18(-0.03-124.06)	2.33(-0.00-4.96)	66.58(-0.03-141.68)	4.07(-0.00-8.99)	117.96(-0.07-261.86)	1.72(-0.00-3.79)	49.54(-0.03-109.77)	-1.32(-1.61--1.03)	-1.02(-1.31--0.73)
Saint Vincent and the Grenadines	1.45(-0.00-3.08)	43.73(-0.01-92.75)	2.13(-0.00-4.54)	62.34(-0.01-132.71)	2.80(-0.00-6.01)	84.85(-0.03-184.30)	2.04(-0.00-4.39)	61.02(-0.02-132.44)	-0.12(-0.36-0.12)	-0.10(-0.31-0.11)

Slovak Republic	95.81(-0.01-207.56)	2821.98(-0.32-6045.48)	1.64(-0.00-3.55)	48.60(-0.01-104.21)	144.20(-0.10-314.38)	3654.99(-2.21-7883.23)	1.55(-0.00-3.37)	41.08(-0.02-88.66)	-0.26(-0.37--0.16)	-0.64(-0.73--0.54)
Socialist Republic of Viet Nam	263.70(-0.05-574.41)	8929.57(-1.83-19598.93)	0.65(-0.00-1.40)	20.86(-0.00-45.70)	873.60(-0.39-1983.93)	28902.84(-12.80-66101.78)	0.85(-0.00-1.90)	25.99(-0.01-59.33)	0.91(0.87-0.95)	0.74(0.70-0.78)
Solomon Islands	1.22(-0.00-2.80)	45.33(-0.01-104.86)	0.81(-0.00-1.85)	25.82(-0.01-59.22)	5.39(-0.00-12.48)	205.38(-0.03-475.51)	1.31(-0.00-3.03)	42.71(-0.01-98.57)	1.68(1.49-1.87)	1.77(1.58-1.96)
State of Eritrea	16.00(-0.00-37.54)	602.91(-0.14-1410.00)	1.22(-0.00-2.83)	37.16(-0.01-86.68)	51.73(-0.01-120.53)	1873.28(-0.41-4392.60)	1.72(-0.00-4.00)	49.16(-0.01-114.09)	1.09(0.96-1.22)	0.87(0.76-0.99)
State of Israel	104.90(-0.08-223.88)	3035.59(-2.68-6540.87)	2.28(-0.00-4.85)	66.35(-0.06-142.30)	173.88(-0.09-374.73)	4197.39(-1.96-9107.15)	1.39(-0.00-2.99)	36.96(-0.02-80.16)	-2.01(-2.25--1.77)	-2.18(-2.38--1.99)
State of Kuwait	4.67(-0.00-9.96)	178.60(-0.03-384.94)	0.65(-0.00-1.39)	19.25(-0.01-41.08)	22.60(-0.01-48.58)	921.04(-0.48-1993.25)	0.60(-0.00-1.29)	19.22(-0.01-41.46)	-0.27(-0.68-0.14)	-0.29(-0.76-0.19)
State of Libya	11.35(-0.00-24.96)	392.37(-0.15-857.88)	0.56(-0.00-1.22)	17.51(-0.01-38.33)	52.77(-0.02-127.33)	1954.13(-0.59-4638.99)	0.86(-0.00-2.05)	27.83(-0.01-66.76)	1.55(1.33-1.78)	1.60(1.37-1.83)
State of Qatar	1.67(-0.00-3.73)	68.68(-0.03-153.15)	1.19(-0.00-2.56)	31.37(-0.01-68.47)	11.25(-0.00-25.07)	463.08(-0.23-1040.02)	1.15(-0.00-2.55)	29.48(-0.01-66.42)	-0.01(-0.36-0.35)	-0.21(-0.42-0.01)
Sultanate of Oman	1.93(-0.00-4.31)	66.69(-0.04-148.84)	0.26(-0.00-0.58)	7.63(-0.00-17.04)	6.49(-0.00-13.94)	238.72(-0.13-522.16)	0.29(-0.00-0.64)	8.39(-0.00-18.26)	0.61(0.28-0.95)	0.57(0.16-0.97)

Swiss Confederation	195.07(-0.14-413.90)	5091.29(-3.64-10848.27)	1.92(-0.00-4.09)	54.19(-0.03-115.54)	204.79(-0.08-449.64)	4594.20(-2.35-9855.37)	1.08(-0.00-2.37)	28.38(-0.02-60.73)	-1.83(-2.00--1.66)	-2.09(-2.31--1.88)
Syrian Arab Republic	34.41(-0.02-75.09)	1304.56(-0.65-2915.34)	0.58(-0.00-1.28)	19.85(-0.01-43.77)	102.55(-0.04-225.36)	3707.40(-1.53-8282.75)	0.72(-0.00-1.57)	24.66(-0.01-54.83)	0.56(0.41-0.70)	0.53(0.37-0.69)
Taiwan (Province of China)	106.42(-0.03-223.73)	4000.91(-0.96-8398.76)	0.63(-0.00-1.32)	21.77(-0.01-45.68)	389.43(-0.20-835.16)	12196.15(-5.75-26282.31)	0.96(-0.00-2.07)	31.76(-0.02-68.74)	1.46(1.24-1.67)	1.25(1.03-1.47)
Togolese Republic	9.80(-0.00-22.65)	364.39(-0.03-844.27)	0.68(-0.00-1.57)	21.74(-0.00-50.15)	41.28(-0.00-95.15)	1492.67(-0.18-3502.79)	0.97(-0.00-2.18)	28.84(-0.00-66.59)	1.25(1.12-1.39)	1.03(0.87-1.18)
Tokelau	0.02(-0.00-0.06)	0.75(-0.00-1.77)	1.96(-0.00-4.65)	60.45(-0.02-140.50)	0.03(-0.00-0.08)	0.97(-0.00-2.26)	2.23(-0.00-5.20)	68.49(-0.04-159.40)	0.41(0.37-0.44)	0.31(0.26-0.36)
Turkmenistan	21.32(-0.00-45.71)	747.28(-0.15-1608.32)	1.05(-0.00-2.25)	33.80(-0.01-72.84)	41.54(-0.02-95.85)	1501.20(-0.74-3481.37)	0.92(-0.00-2.12)	30.98(-0.02-71.68)	0.02(-0.44-0.49)	0.22(-0.24-0.69)
Tuvalu	0.11(-0.00-0.27)	3.96(-0.00-9.36)	1.75(-0.00-3.99)	53.55(-0.03-124.92)	0.21(-0.00-0.48)	6.54(-0.00-15.24)	2.03(-0.00-4.69)	59.56(-0.02-138.17)	0.41(0.34-0.48)	0.28(0.20-0.35)
Ukraine	1332.65(-0.35-2865.85)	41145.57(-5.85-87710.28)	1.93(-0.00-4.14)	60.89(-0.01-129.74)	1046.97(-0.47-2411.59)	29353.81(-13.74-68982.38)	1.40(-0.00-3.23)	41.51(-0.02-98.06)	-1.54(-1.77--1.30)	-1.78(-2.03--1.54)
Union of the Comoros	2.22(-0.00-4.85)	78.75(-0.01-172.62)	1.05(-0.00-2.32)	32.48(-0.00-71.20)	6.97(-0.00-16.13)	235.56(-0.02-558.59)	1.35(-0.00-3.11)	40.20(-0.00-94.92)	0.61(0.49-0.73)	0.41(0.24-0.58)

United Arab Emirates	5.97(-0.00-12.94)	228.38(-0.01-497.62)	1.13(-0.00-2.43)	32.02(-0.00-68.34)	45.61(-0.02-104.36)	1795.80(-1.11-4096.99)	1.20(-0.00-2.71)	29.79(-0.01-67.43)	2.00(1.36-2.66)	1.14(0.62-1.67)
United Kingdom of Great Britain and Northern Ireland	2403.01(-0.92-5105.53)	61421.05(-24.53-129959.34)	2.82(-0.00-5.98)	78.76(-0.03-167.41)	1880.85(-0.55-4064.28)	43386.25(-11.97-92149.90)	1.44(-0.00-3.08)	38.85(-0.01-82.44)	-2.29(-2.38--2.21)	-2.39(-2.46--2.32)
United Mexican States	383.86(-0.13-820.89)	13283.88(-4.58-28425.39)	0.84(-0.00-1.80)	25.88(-0.01-55.50)	1290.85(-0.46-2784.81)	42716.49(-14.68-92195.85)	0.99(-0.00-2.13)	31.42(-0.01-67.77)	0.29(0.19-0.39)	0.36(0.26-0.46)
United Republic of Tanzania	130.74(-0.02-291.81)	4487.01(-0.88-9916.87)	1.19(-0.00-2.69)	34.84(-0.01-77.76)	395.39(-0.08-911.25)	13672.20(-3.05-31606.75)	1.44(-0.00-3.33)	41.68(-0.01-96.02)	0.61(0.48-0.75)	0.54(0.40-0.68)
United States of America	6722.65(-3.07-14390.77)	194436.50(-92.98-415220.87)	2.18(-0.00-4.66)	66.24(-0.03-141.25)	7309.40(-3.14-15670.99)	194577.63(-88.97-416871.17)	1.29(-0.00-2.75)	37.87(-0.02-81.15)	-1.85(-1.92--1.78)	-1.93(-2.00--1.86)
United States Virgin Islands	1.90(-0.00-4.13)	62.80(-0.02-133.38)	2.24(-0.00-4.94)	64.21(-0.02-137.60)	2.47(-0.00-5.87)	66.20(-0.03-160.37)	1.58(-0.00-3.75)	47.31(-0.02-113.39)	-0.84(-1.00--0.68)	-0.66(-0.80--0.52)

Table S4: Age-standardized disability-adjusted life year rates of breast cancer attributed to diet high in red meat with frontier analysis across 204 countries and territories.

**Location**	**SDI**	**Rate of DALYs**	**Frontier DALYs**	**Effective difference**
Afghanistan	0.337199998	33.11548956	5.102002342	28.01348722
Albania	0.706849791	22.17930192	5.092528689	17.08677323
Algeria	0.659500924	17.16140984	5.116883943	12.0445259
American Samoa	0.723727533	82.73394408	5.11278103	77.62116305

Andorra	0.869444113	35.88995517	5.095104117	30.79485105
Angola	0.453721949	42.0930265	5.111421113	36.98160539
Antigua and Barbuda	0.749886887	62.24502337	5.095452097	57.14957127
Argentina	0.723122973	49.27641833	5.119618787	44.15679954
Armenia	0.701833194	42.94521236	5.11227887	37.83293349
Australia	0.844252814	32.17192682	5.09482981	27.07709701
Austria	0.853837004	32.71149162	5.120610478	27.59088114
Azerbaijan	0.694851274	33.41488938	5.111131385	28.303758
Bahamas	0.805020668	85.18685725	5.106825011	80.08003224
Bahrain	0.753043204	38.5447659	5.09759881	33.44716709
Bangladesh	0.492420885	9.249860951	5.113398913	4.136462038
Barbados	0.746748764	73.55531917	5.111691664	68.44362751
Belarus	0.784484711	35.5768006	5.099703147	30.47709745
Belgium	0.853654016	39.91814311	5.101791442	34.81635167
Belize	0.610229002	27.6554988	5.10598618	22.54951262
Benin	0.373486574	21.51274748	5.099827447	16.41292003
Bermuda	0.821365422	41.64871107	5.082853414	36.56585766
Bhutan	0.473062378	13.61281002	5.08584573	8.52696429
Bolivia (Plurinational State of)	0.599010799	38.56215467	5.138348014	33.42380666
Bosnia and Herzegovina	0.723077893	34.76647886	5.121170321	29.64530854
Botswana	0.642721629	45.23828441	5.128042683	40.11024173
Brazil	0.653043887	39.23329673	5.114697835	34.11859889
Brunei Darussalam	0.810234367	41.31153242	5.104142781	36.20738964
Bulgaria	0.768150939	51.52146063	5.101024416	46.42043621
Burkina Faso	0.285118402	45.27782241	5.290578973	39.98724344
Burundi	0.289374365	12.26166136	5.293890001	6.967771359
Cabo Verde	0.533534539	28.98505951	5.113967769	23.87109174
Cambodia	0.473621491	46.51682747	5.13398088	41.38284659
Cameroon	0.479691223	36.19028998	5.110768374	31.07952161

Canada	0.87317068	32.95223897	5.089627332	27.86261164
Central African Republic	0.30916769	41.31732777	5.320196762	35.99713101
Chad	0.240436019	24.46654615	5.657859193	18.80868696
Chile	0.771514716	26.58542455	5.111905072	21.47351948
China	0.72162976	19.9451094	5.147360552	14.79774885
Colombia	0.655442913	33.49034035	5.102529841	28.38781051
Comoros	0.475978688	40.19513389	5.09334673	35.10178716
Congo	0.583075236	61.00959552	5.102510846	55.90708467
Cook Islands	0.779109955	78.82533476	5.098444766	73.72688999
Costa Rica	0.700340477	37.62098956	5.083730472	32.53725909
Côte d'Ivoire	0.425941883	42.35279527	5.088856148	37.26393912
Croatia	0.798341027	38.0337141	5.106327313	32.92738679
Cuba	0.668729864	35.6792569	5.12026048	30.55899642
Cyprus	0.835630545	40.77765103	5.110205144	35.66744589
Czechia	0.828450433	32.68335067	5.112242397	27.57110827
Democratic People's Republic of Korea	0.569854634	25.44488324	5.123488058	20.32139518
Democratic Republic of the Congo	0.383179849	18.04195169	5.107683456	12.93426823
Denmark	0.896424204	36.8045767	5.132721152	31.67185555
Djibouti	0.487958371	45.22867484	5.080062917	40.14861192
Dominica	0.746967185	61.3979879	5.116037866	56.28195003
Dominican Republic	0.619388201	29.49988419	5.109995743	24.38988845
Ecuador	0.661017053	27.19110829	5.100658786	22.0904495
Egypt	0.606787094	36.15227135	5.134296492	31.01797486
El Salvador	0.563775188	27.43470733	5.100406563	22.33430077
Equatorial Guinea	0.657857456	56.1750389	5.090164322	51.08487458
Eritrea	0.403863943	49.15796689	5.093973651	44.06399324
Estonia	0.844917787	32.83130405	5.122675502	27.70862855
Eswatini	0.585459713	60.08955881	5.094520294	54.99503852

Ethiopia	0.358823295	33.28089036	5.101966758	28.1789236
Fiji	0.675051631	81.05185594	5.097317384	75.95453856
Finland	0.859831368	31.94820489	5.127263729	26.82094116
France	0.838364875	41.25235434	5.080020838	36.1723335
Gabon	0.634691393	56.8580179	5.106873848	51.75114405
Gambia	0.40971416	15.7515654	5.09807578	10.65348962
Georgia	0.732473604	63.4763245	5.094921295	58.3814032
Germany	0.902957091	40.18485902	5.088603613	35.09625541
Ghana	0.56493039	38.47658743	5.128911574	33.34767586
Greece	0.791854408	43.43888744	5.07806933	38.36081811
Greenland	0.826210336	34.46398372	5.08820586	29.37577786
Grenada	0.668993028	59.897216	5.124784729	54.77243127
Guam	0.803982203	33.32858251	5.116630925	28.21195159
Guatemala	0.539972424	18.17638799	5.131953857	13.04443413
Guinea	0.336401293	30.83788593	5.109990882	25.72789505
Guinea-Bissau	0.353109621	45.27511271	5.091798538	40.18331417
Guyana	0.650812335	41.19776738	5.089935848	36.10783153
Haiti	0.448278285	49.61869908	5.117664479	44.5010346
Honduras	0.513037248	27.92290933	5.104246833	22.8186625
Hungary	0.790754768	43.18530358	5.122288311	38.06301527
Iceland	0.87636168	34.64970572	5.089975272	29.55973045
India	0.575401649	10.06837028	5.095137195	4.973233085
Indonesia	0.656868336	29.03565746	5.113765847	23.92189161
Iran (Islamic Republic of)	0.697207398	20.89219576	5.118245646	15.77395011
Iraq	0.662626231	27.45653829	5.110528853	22.34600944
Ireland	0.87375385	36.41602651	5.119571441	31.29645507
Israel	0.809011652	36.96089019	5.103319852	31.85757034
Italy	0.805773534	37.58446772	5.127524089	32.45694363
Jamaica	0.683263064	58.69539402	5.135773617	53.5596204

Japan	0.871241813	24.19270601	5.092218942	19.10048707
Jordan	0.725307227	31.14464095	5.07835796	26.06628299
Kazakhstan	0.725144495	30.71466712	5.136121349	25.57854577
Kenya	0.523768077	41.69523414	5.099798151	36.59543599
Kiribati	0.527186583	69.63537025	5.13881475	64.4965555
Kuwait	0.846651055	19.21677552	5.114842083	14.10193344
Kyrgyzstan	0.603979328	27.40555314	5.107450303	22.29810284
Lao People's Democratic Republic	0.489136091	40.12518772	5.105063015	35.02012471
Latvia	0.830663516	40.32667802	5.106851251	35.21982677
Lebanon	0.744746351	45.41993674	5.088162852	40.33177389
Lesotho	0.510393066	60.17146962	5.108935685	55.06253393
Liberia	0.352442452	22.5698453	5.098133171	17.47171213
Libya	0.725771399	27.83063605	5.110658424	22.71997763
Lithuania	0.856484049	39.10642357	5.109727967	33.9966956
Luxembourg	0.884428955	33.85749944	5.092981581	28.76451786
Madagascar	0.400246943	38.27814811	5.110968432	33.16717968
Malawi	0.384553634	33.94203843	5.070819642	28.87121879
Malaysia	0.742523828	54.18568938	5.120668721	49.06502066
Maldives	0.650886627	9.199557934	5.11917626	4.080381674
Mali	0.268579941	35.12999459	5.280875421	29.84911917
Malta	0.801585034	41.7203871	5.135266492	36.58512061
Marshall Islands	0.574091128	64.29843551	5.101420766	59.19701474
Mauritania	0.4989451	36.80909441	5.084168783	31.72492563
Mauritius	0.718260446	50.60560714	5.106380397	45.49922674
Mexico	0.664575304	31.42283392	5.068895106	26.35393881
Micronesia (Federated States of)	0.587534967	70.56432774	5.096651427	65.46767631
Monaco	0.908262831	76.45568908	5.094628101	71.36106098
Mongolia	0.617621565	13.79914833	5.104170887	8.694977443
Montenegro	0.795800584	54.17939098	5.097102376	49.0822886

Morocco	0.562698301	22.06067625	5.138719408	16.92195684
Mozambique	0.326462614	34.72314162	5.299743832	29.42339779
Myanmar	0.53390084	44.38408734	5.08595196	39.29813538
Namibia	0.617564872	73.00851673	5.08463772	67.92387901
Nauru	0.625177834	86.55176518	5.10101289	81.45075229
Nepal	0.433174635	20.40683803	5.1226082	15.28422983
Netherlands	0.888464256	40.86063997	5.111185986	35.74945398
New Zealand	0.849442499	39.69896963	5.099692654	34.59927698
Nicaragua	0.523958472	17.50617848	5.102454008	12.40372447
Niger	0.168072774	19.86623402	17.48350451	2.382729506
Nigeria	0.503390833	52.38808251	5.111809016	47.27627349
Niue	0.72622205	71.67370971	5.114249639	66.55946007
North Macedonia	0.750629703	49.27876281	5.107668774	44.17109404
Northern Mariana Islands	0.771535213	54.87722521	5.092916507	49.7843087
Norway	0.91613281	26.53018663	5.130017459	21.40016917
Oman	0.773391602	8.392757713	5.090244173	3.30251354
Pakistan	0.504028689	58.27572728	5.090530076	53.1851972
Palau	0.754046931	74.46518426	5.115910516	69.34927374
Palestine	0.631011665	39.16003731	5.097222217	34.06281509
Panama	0.708864828	33.06920985	5.13331031	27.93589954
Papua New Guinea	0.417797443	40.22749807	5.139714451	35.08778362
Paraguay	0.635718099	38.08772098	5.095889837	32.99183114
Peru	0.662054037	24.82607217	5.104289727	19.72178244
Philippines	0.651219329	54.32672414	5.101695088	49.22502905
Poland	0.812042809	41.90768906	5.112449882	36.79523918
Portugal	0.744151851	34.94575971	5.111296428	29.83446328
Puerto Rico	0.825525847	35.72966203	5.117432768	30.61222926
Qatar	0.846860584	29.47763249	5.095910485	24.381722
Republic of Korea	0.886675267	16.0379675	5.099662836	10.93830466

Republic of Moldova	0.732214875	40.53187183	5.095622632	35.4362492
Romania	0.768453864	43.86645004	5.111263306	38.75518673
Russian Federation	0.808536005	42.12015985	5.101162888	37.01899696
Rwanda	0.435588706	40.97432429	5.101850275	35.87247401
Saint Kitts and Nevis	0.754987055	57.01752347	5.106201976	51.91132149
Saint Lucia	0.672509735	49.53666894	5.113431627	44.42323731
Saint Vincent and the Grenadines	0.637195963	61.01845845	5.116594099	55.90186435
Samoa	0.593392769	43.96414983	5.118064513	38.84608532
San Marino	0.888005474	24.1163921	5.108336773	19.00805533
Sao Tome and Principe	0.505413747	17.2430862	5.104890579	12.13819562
Saudi Arabia	0.815143493	18.42543462	5.116299506	13.30913511
Senegal	0.408054193	33.19598728	5.096649719	28.09933756
Serbia	0.792416294	54.23441036	5.096385655	49.13802471
Seychelles	0.730150775	39.77762636	5.085150726	34.69247563
Sierra Leone	0.358665881	19.33760179	5.111324865	14.22627693
Singapore	0.856097766	23.76611581	5.153077097	18.61303871
Slovakia	0.81061053	41.07520198	5.092691077	35.9825109
Slovenia	0.842430731	30.44936156	5.094886873	25.35447469
Solomon Islands	0.429360316	42.70672098	5.127824186	37.57889679
Somalia	0.077688109	32.76574901	31.47030909	1.295439918
South Africa	0.679626598	53.50886195	5.063415993	48.44544596
South Sudan	0.278371125	31.7590003	5.282073039	26.47692726
Spain	0.769283698	29.09589956	5.120385474	23.97551409
Sri Lanka	0.701534935	9.635487104	5.134329698	4.501157406
Sudan	0.541949735	17.27274376	5.077164606	12.19557915
Suriname	0.633665739	34.83118728	5.119306757	29.71188052
Sweden	0.886880299	26.56748	5.079766347	21.48771365
Switzerland	0.933059111	28.38199575	5.095617127	23.28637862
Syrian Arab Republic	0.623004075	24.65795031	5.090680165	19.56727015

Taiwan (Province of China)	0.874747053	31.76322132	5.09883145	26.66438987
Tajikistan	0.541511187	23.65997254	5.104704504	18.55526804
Thailand	0.682547933	38.13646629	5.112545106	33.02392118
Timor-Leste	0.444667619	27.42849335	5.118186869	22.31030648
Togo	0.408533695	28.84038528	5.109926268	23.73045901
Tokelau	0.686425621	68.48773894	5.132092394	63.35564655
Tonga	0.626349936	79.61045986	5.104164751	74.50629511
Trinidad and Tobago	0.768763254	55.13906849	5.118738719	50.02032977
Tunisia	0.682432216	24.8288644	5.104739252	19.72412515
Turkey	0.712692673	28.45491057	5.133064603	23.32184597
Turkmenistan	0.682160776	30.97891171	5.104095927	25.87481578
Tuvalu	0.576620529	59.56368631	5.117000451	54.44668586
Uganda	0.423261181	57.49705804	5.142243116	52.35481492
Ukraine	0.760773913	41.51020662	5.142355054	36.36785157
United Arab Emirates	0.849317734	29.78636403	5.098453063	24.68791097
United Kingdom	0.859000182	38.8511706	5.130966785	33.72020382
United Republic of Tanzania	0.446568273	41.67729403	5.106894937	36.57039909
United States of America	0.862448354	37.87289765	5.105844006	32.76705364
United States Virgin Islands	0.821830853	47.31484121	5.107516831	42.20732438
Uruguay	0.719283445	62.3183245	5.104870849	57.21345365
Uzbekistan	0.662621694	28.02709881	5.12118253	22.90591628
Vanuatu	0.473100706	44.32085788	5.107550624	39.21330726
Venezuela (Bolivarian Republic of)	0.596513059	45.8708288	5.10667406	40.76415474
Viet Nam	0.627933721	25.99461179	5.116209933	20.87840186
Yemen	0.450376375	15.71132061	5.097816496	10.61350411
Zambia	0.505948954	64.58049699	5.126215586	59.4542814
Zimbabwe	0.473819486	64.99091745	5.10078541	59.89013204

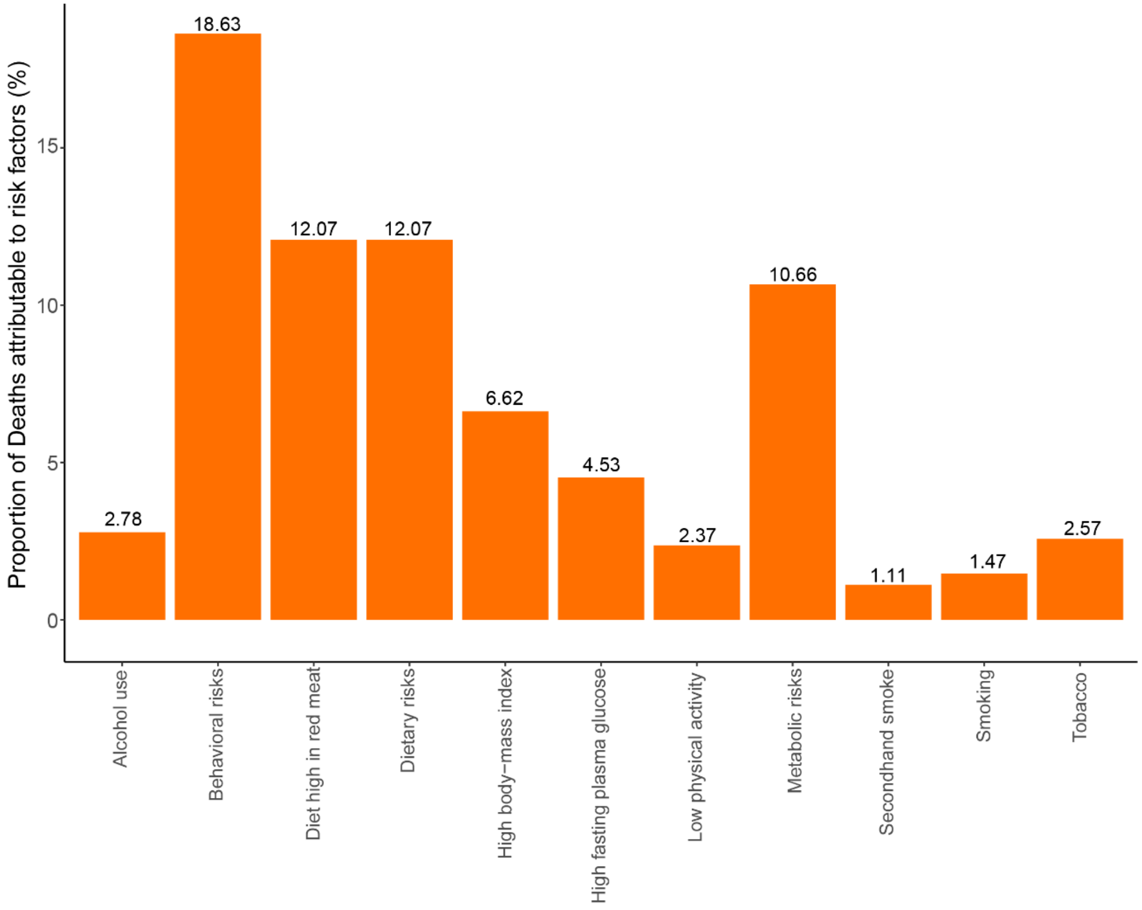

Figure S1: Proportion of attributable risk factors for global breast cancer deaths in 2021.

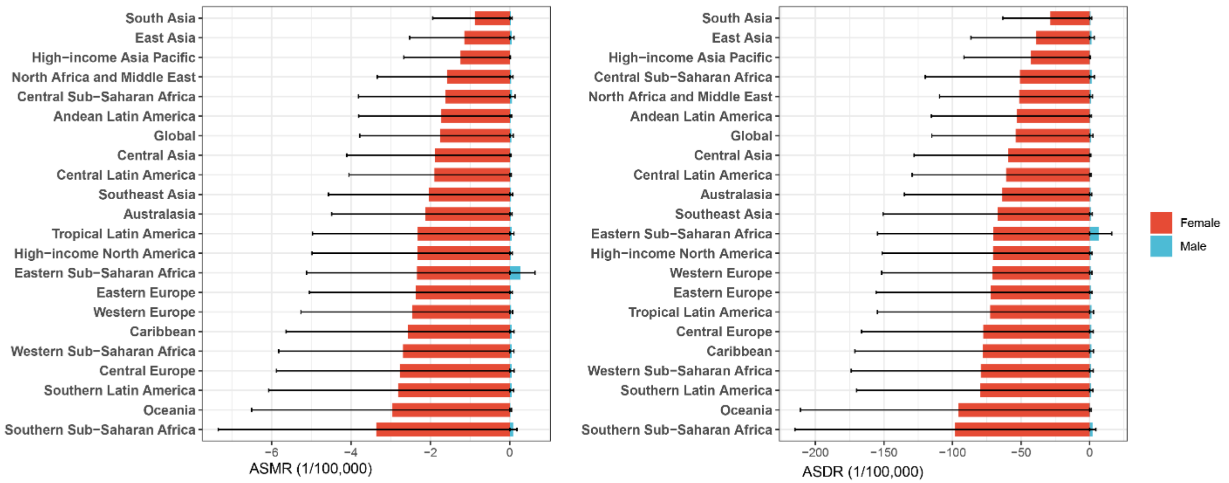

Figure S2: Gender differences in age-standardized mortality rates and age-standardized disability-adjusted life year rates for breast cancer attributed to diet high in red meat in 2021.

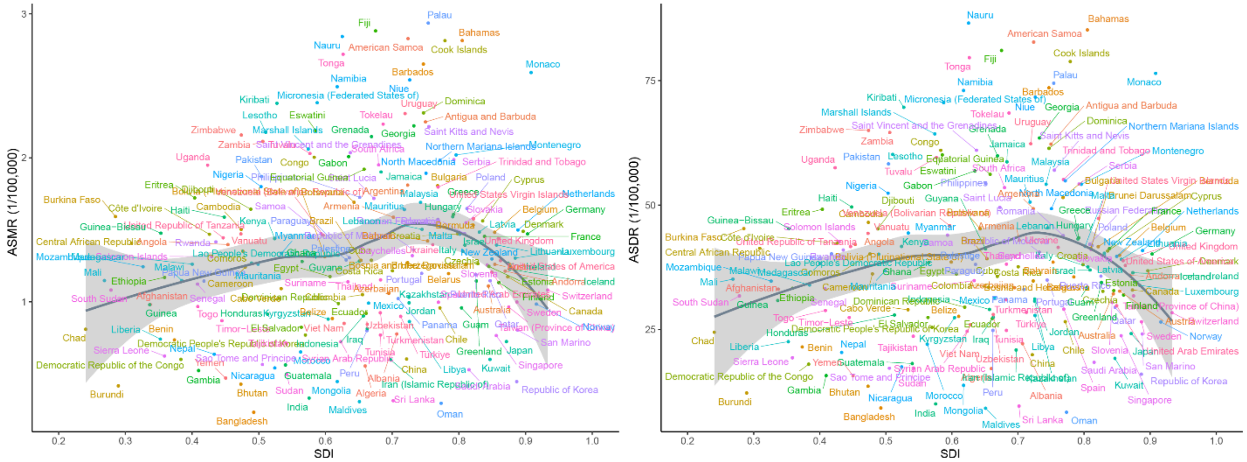

Figure S3: Age-standardized mortality rates and age-standardized disability-adjusted life year rates for breast cancer attributed to diet high in red meat across 204 countries and territories in 2021 by sociodemographic index.
